# Chemical Compounds and Antioxidant Activity of *Forsythia suspensa* Leaves Black Tea

**DOI:** 10.3390/molecules31101687

**Published:** 2026-05-16

**Authors:** Shuheng Wang, Qi Du, Junwen Ma, Xin Yuan, Shifei Li, Xiaoxia Gao, Liwei Zhang

**Affiliations:** 1 Key Laboratory of Chemical Biology and Molecular Engineering of Ministry of Education, Institute of Molecular Science, Shanxi University, No. 92, Wucheng Road, Taiyuan 030006, China; 202214301003@email.sxu.edu.cn (S.W.);; 2 Modern Research Center for Traditional Chinese Medicine, Shanxi University, No. 92, Wucheng Road, Taiyuan 030006, China; 3 Key Laboratory of Research and Utilization of Bioactive Components in Famous Shanxi Medicinal Materials, Shanxi University, No. 92, Wucheng Road, Taiyuan 030006, China

**Keywords:** *Forsythia suspensa* leaves black tea, lignan aglycones, phillygenin, antioxidant activity, fermented herbal tea

## Abstract

*Forsythia suspensa* leaves black tea (FSLBT) is a fermented herbal tea traditionally consumed in Northern China, yet its bioactive constituents and antioxidant basis remain insufficiently understood. In this study, a phytochemical investigation of FSLBT led to the isolation and structural identification of nine compounds, including five lignans and four pentacyclic triterpenic acids, whose structures were elucidated by ^1^H-NMR, ^13^C-NMR, and HRESIMS spectral analysis. Notably, epipinoresinol and pinoresinol monomethyl ether were isolated from *Forsythia suspensa* leaves (FSL) for the first time. Among the isolated lignans, phillygenin, epipinoresinol, pinoresinol monomethyl ether, and pinoresinol were further evaluated for antioxidant activity using DPPH•, ABTS•+, and FRAP assays. The four lignans exhibited concentration-dependent antioxidant activities, with *IC*_50_ (half maximal inhibitory concentration) values ranging from 20.32 to 46.40 μg/mL for DPPH• scavenging and 37.29 to 72.71 μg/mL for ABTS•+ scavenging, while FRAP *EC*_50_ (half maximal effective concentration) values ranged from 1.53 to 1.90 mg/mL. Quantitative HPLC analysis showed that the contents of phillygenin, epipinoresinol, pinoresinol monomethyl ether, and pinoresinol in FSLBT were 3.48 ± 1.12 wt%, 0.39 ± 0.21 wt%, 0.26 ± 0.20 wt%, and 0.18 ± 0.07 wt%, respectively. These results indicate that FSLBT is enriched in lignan aglycones, particularly phillygenin, and that these major lignans possess measurable chemical antioxidant activities, providing a phytochemical basis for further investigation of the functional properties of this fermented herbal tea.

## 1. Introduction

*Forsythia suspensa* (Thunb.) Vahl (Oleaceae) is a well-known medicinal plant widely used in traditional Chinese medicine (TCM) [1]. The fruits of *F. suspensa* have mainly been used for the treatment of gonorrhea, erysipelas, inflammation, and ulcers [2,3]. The FSL, documented in *Bencao Gangmu* (1593 CE), have been used for over 400 years to clear heat and remove fire, as well as a functional tea in Chinese folk medicine, particularly in Shanxi, Hebei, and Shaanxi provinces [4,5]. Phytochemical investigations of FSL have identified abundant phenylethanoid glycosides, lignans, and triterpenoid acids [6]. These compounds exhibit a wide range of bioactivities, including antioxidant, anti-inflammatory, hepatoprotective, hypolipidemic, and anti-obesity effects [7,8,9].

*Forsythia suspensa* leaves tea (FSLT) is typically categorized into two types based on processing methods: *F. suspensa* leaves green tea (FSLGT) and FSLBT. FSLGT, prepared mainly by fixation of fresh leaves, largely preserves the chemical profile of the raw materials [10,11,12,13,14]. In contrast, FSLBT is produced through spontaneous fermentation in which endogenous enzymes and accompanying biochemical reactions may alter the phytochemical composition. Such processing-induced changes may be particularly important for lignan constituents, because glycosylated precursors can potentially undergo hydrolysis or related conversions during fermentation [15,16].

Previous studies on *F. suspensa* leaves have largely focused on non-fermented leaves and general tea infusions, reporting abundant triterpenoids, phenolic compounds, flavonoids, and lignans in these preparations, whereas the phytochemical composition of FSLBT has received much less attention [5,17]. In contrast, the phytochemical composition of FSLBT and the chemical antioxidant activity of its major lignan aglycones remain poorly characterized. Clarifying these issues is important for understanding the chemical basis of this traditional fermented herbal tea and for supporting its quality evaluation.

Therefore, the present study aimed to investigate the major constituents of FSLBT through conventional phytochemical isolation and structural identification, to evaluate the chemical antioxidant activities of representative lignan aglycones using DPPH•, ABTS•+, and FRAP assays, and to quantify these major lignan aglycones by HPLC. This study focuses on the predominant lignan aglycones and a subset of triterpenic acids in FSLBT and provides experimental data supporting the contribution of these compounds to its chemical antioxidant potential.

## 2. Results

### 2.1. Identification of Isolated Compounds

**Figure 1:**
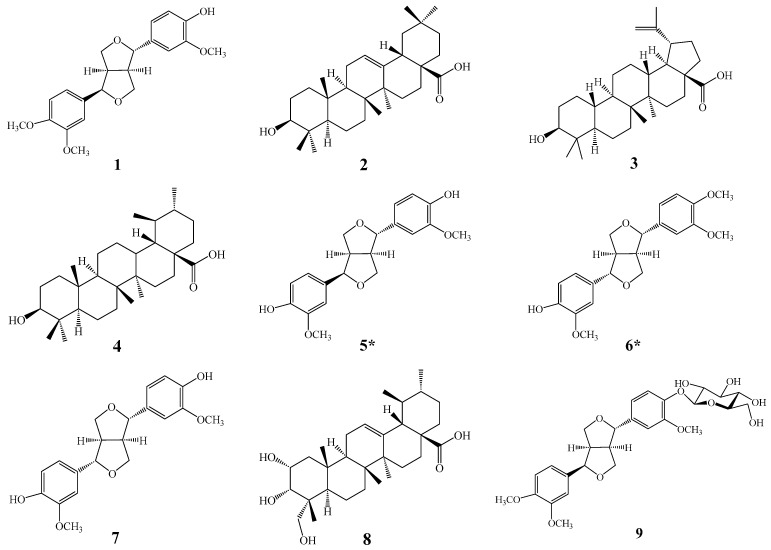
Chemical structures of **1**–**9**. (* represents the first compound isolated from leaves of *F. suspensa*).

The ethanolic extract of FSLBT was separated by D101 macroporous resin, repeated silica gel CC, Sephadex LH-20 and semipreparative HPLC to afford nine compounds (**1**–**9**) (Figure 1). These compounds include five lignans and four pentacyclic triterpenic acids: Phillygenin (**1**), Betulinic acid (**2**), Oleanolic acid (**3**), Ursolic acid (**4**), Epipinoresinol (**5**), Pinoresinol monomethyl ether (**6**), Pinoresinol (**7**), Esculentic acid (**8**), and Phillyrin (**9**).

All isolates were characterized by high-resolution electrospray ionization mass spectrometry (HRESIMS) using a Thermo Scientific Q Exactive LC-MS/MS system and by ^1^H NMR and ^13^C-NMR spectroscopy (Bruker, 600 MHz for ^1^H, 125/150 MHz for ^13^C). Compounds **1**–**4** and **7**–**9** have been previously reported from *Forsythia suspensa* leaves and were identified by comparison with the corresponding literature data [18,19,20,21,22,23,24,25], whereas compounds **5** and **6** were isolated from FSL for the first time.

The four major lignan aglycones (Phillygenin, Epipinoresinol, Pinoresinol monomethyl ether, and Pinoresinol) were quantified and their detailed ^1^H and ^13^C NMR chemical shifts are summarized in Table 1. These data confirm the identities of the active lignans in FSLBT. For completeness, full NMR and HRESIMS spectra of all nine compounds, including the previously reported triterpenic acids and lignans, are provided in the Supplementary Materials (Figures S3–S38; Tables S1 and S2).

### 2.2. Antioxidant Activity of the Four Lignans

**Figure 2:**
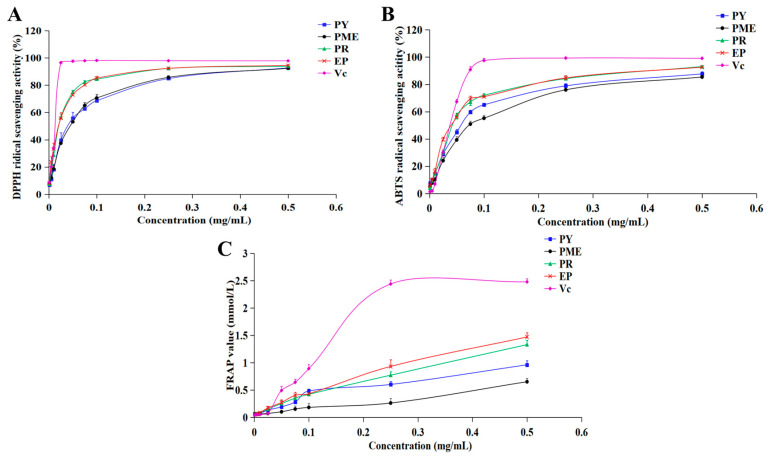
Antioxidant activity of four lignan compounds (PY: Phillygenin; PME: Pinoresinol monomethyl ether; PR: Pinoresinol; EP: Epipinoresinol; Vc: Vitamin C). ((**A**): DPPH•; (**B**): ABTS•+; (**C**): FRAP).

The chemical antioxidant activities of four lignans isolated from FSLBT, namely phillygenin, pinoresinol monomethyl ether, epipinoresinol, and pinoresinol, were evaluated using DPPH, ABTS, and FRAP assays. As shown in Figure 2A–C, all four compounds exhibited concentration-dependent activities in the three assays.

As shown in Figure 2A, all tested lignans were found to have significant DPPH• scavenging activity in a concentration-dependent manner. Table 2 summarizes the *IC*_50_ values (μg/mL): phillygenin, 43.26 ± 4.46; pinoresinol monomethyl ether, 46.40 ± 1.22; epipinoresinol, 20.32 ± 1.34; pinoresinol, 20.98 ± 0.73. The results showed that epipinoresinol and pinoresinol have a stronger ability to scavenge DPPH• than phillygenin and pinoresinol monomethyl ether.

The ABTS•+ radical cation scavenging activities of the tested lignans are depicted in Figure 2B. The results showed that all tested lignans exhibited significant ABTS•+ scavenging activity in a concentration-dependent manner. *IC*_50_ values (μg/mL) were quantified as follows (Table 2): phillygenin, 54.27 ± 0.36; pinoresinol monomethyl ether, 72.71 ± 0.58; epipinoresinol, 37.29 ± 1.05; pinoresinol, 42.07 ± 0.55. The results showed that epipinoresinol and pinoresinol have a stronger ability to scavenge ABTS•+ than phillygenin and pinoresinol monomethyl ether. Among them, the scavenging ability of epipinoresinol was close to the ABTS•+ scavenging activity of V_C_ (36.51 ± 0.44 μg/mL).

As illustrated in Figure 2C, all tested lignans exhibited high reducing power activity. The reducing power of the lignans showed a positive correlation with concentration. Table 2 summarizes the *EC*_50_ values (mg/mL): phillygenin, 1.63 ± 0.02; pinoresinol monomethyl ether, 1.90 ± 0.07; epipinoresinol, 1.53 ± 0.01; and pinoresinol, 1.58 ± 0.03. The four lignans exhibited comparable reducing power.

Overall, epipinoresinol and pinoresinol showed lower *IC*_50_ values than phillygenin and pinoresinol monomethyl ether in both the DPPH and ABTS assays, whereas the four compounds exhibited relatively comparable reducing power in the FRAP assay.

### 2.3. Quantification of Major Lignan Aglycones in FSLBT

**Figure 3:**
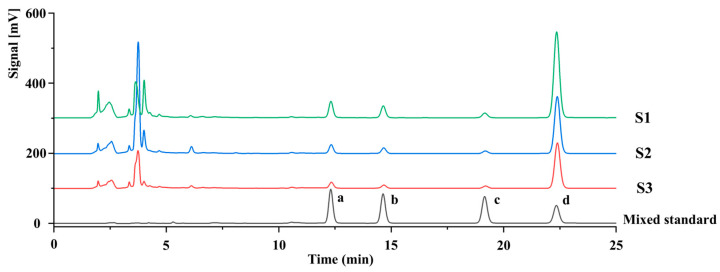
Chromatograms of compounds in the FSLBT samples and standard solution at 280 nm. (pinoresinol (a), epipinoresinol (b), pinoresinol monomethyl ether (c) and phillygenin (d)).

Representative HPLC chromatograms of the mixed reference standards and three batches of FSLBT samples are shown in Figure 3. Four major lignan aglycones, namely pinoresinol, epipinoresinol, pinoresinol monomethyl ether, and phillygenin, were detected and quantified in FSLBT. As summarized in Table 3, the contents of phillygenin, pinoresinol, epipinoresinol, and pinoresinol monomethyl ether in FSLBT were 3.48 ± 1.12%, 0.39 ± 0.21%, 0.26 ± 0.20%, and 0.18 ± 0.07% (mean ± SD, *n* = 3 batches), respectively. Among the four compounds, phillygenin was the most abundant lignan aglycone in FSLBT. These results indicate that FSLBT is characterized by a relatively high abundance of lignan aglycones, especially phillygenin. The HPLC method was validated for linearity, precision, repeatability, stability, and recovery for all four lignan standards. Calibration curves showed good linearity (R^2^ > 0.999, Figure S2). Precision, repeatability, stability, and recovery experiments demonstrated that the method was accurate and reproducible. Detailed validation data are provided in the Supplementary Material (Figure S2, Tables S3–S9).

## 3. Discussion

Natural plant constituents obtained through isolation, structural elucidation, and purification—including lignans, phenolic compounds, and other secondary metabolites—frequently exhibit significant antioxidant potential [26,27,28,29]. Among the four major lignan aglycones tested, epipinoresinol and pinoresinol exhibited the strongest DPPH and ABTS radical-scavenging activities, with *IC*_50_ values of 20.32 and 20.98 μg/mL for DPPH, and 37.29 and 42.07 μg/mL for ABTS, respectively. Phillygenin and pinoresinol monomethyl ether showed comparatively moderate activities. FRAP assay results were less discriminating, but all four lignans displayed dose-dependent reducing power. These results may be explained by the fact that epipinoresinol and pinoresinol contain two ortho-methoxy-hydroxyl pairs, whereas phillygenin and pinoresinol monomethyl ether possess only one. Previous studies have shown that the chemical antioxidant activity of lignans is closely related to their phenolic hydroxyl groups and other oxygen-containing substituents [30]. Phenolic hydroxyl groups (-OH) critically determine antioxidant efficacy [31]. The presence of methoxy groups at the ortho-position relative to hydroxyl groups significantly enhances antioxidant activity [32,33]. The quantity of these conjugated systems exhibits a significant positive correlation with radical scavenging activity. Consistent with this mechanism, epipinoresinol and pinoresinol exhibited twofold higher DPPH• scavenging activity than phillygenin and pinoresinol monomethyl ether.

Representative HPLC analysis of three independent batches of FSLBT showed that the contents of phillygenin, pinoresinol, epipinoresinol, and pinoresinol monomethyl ether varied across batches, ranging from 2.55–4.74%, 0.22–0.63%, 0.09–0.48%, and 0.12–0.26%, respectively. Despite these batch-to-batch variations, phillygenin remained the predominant lignan aglycone in all batches. This demonstrates that while minor fluctuations exist due to differences in leaf harvest dates or other factors, the overall phytochemical profile and the relative abundance of major lignans are consistent.

The HPLC results showed that FSLBT contained four major lignan aglycones, among which phillygenin was the predominant compound. While fermentation may contribute to the accumulation of these lignans, the present study did not directly compare fermented and unfermented leaves. Previous studies have suggested that fermentation of herbal teas can generate or enrich secondary metabolites that are absent or less abundant in unfermented materials [34,35,36]. Phillyrin, a major lignan glycoside in *F. suspensa* leaves, is a plausible precursor of phillygenin, and glycosylated forms of pinoresinol monomethyl ether and epipinoresinol have also been reported in unfermented leaves [37,38]. Therefore, the observed high abundance of lignan aglycones in FSLBT may reflect a combination of natural presence and possible fermentation-related transformation, though direct experimental evidence for enzymatic conversion was not obtained in this study.

It should be noted that the phillygenin content reported in the literature (~30%) refers to enriched crude extracts of *F. suspensa* leaves [14], whereas in the present study, the quantitative HPLC analysis was performed directly on commercially obtained FSLBT leaf material. The phillygenin content in our three independent batches (2.55–4.74%) is therefore naturally lower than that reported for enriched extracts. Nevertheless, phillygenin remains the predominant lignan aglycone in FSLBT, providing a reliable characterization of its lignan profile.

The pharmacological, toxicological, and pharmacokinetic properties of phillygenin have been comprehensively reviewed, and efficient extraction methods for this and related lignans from *F. suspensa* leaves have been reported [39,40]. Building on these previous studies, the abundant accumulation of lignan aglycones, especially phillygenin, in FSLBT highlights the tea as a valuable source of these compounds and provides a solid basis for their quality evaluation. Combined phytochemical identification and quantitative HPLC analysis further support future quality control of fermented *F. suspensa* leaf products.

## 4. Materials and Methods

### 4.1. Plant Materials

The FSLBT samples utilized in this study were provided by Shanxi Guanlin Agricultural Science and Technology Corporation. (Pingding County, Yangquan, China). All samples were processed using traditional black tea fermentation methods prior to purchase and were supplied as dried leaf material. Three independent batches were obtained (harvested on 1 May (S1), 15 May (S2), and 16 June (S3), 2025) and used for quantitative analysis and compound isolation. The samples were stored at 4 °C in sealed containers until extraction.

### 4.2. Extraction and Isolation

Powdered FSLBT (5 kg) was extracted three times with 70% ethanol under reflux (2 h per cycle). The combined extracts were filtered and concentrated under reduced pressure to afford a dark brown crude extract (1.0 kg). A portion of the crude extract (150 g) was subjected to D101 macroporous resin column chromatography and eluted stepwise with ethanol-water mixtures (0%, 30%, 50%, 70%, and 95%, *v*/*v*) to yield four fractions (Fr. A-Fr. D). Fraction D (60 g) was recrystallized from methanol to afford compound **1** (15 g). The mother liquor (45 g) was further separated by silica gel column chromatography using a petroleum ether-ethyl acetate gradient (100:0 to 0:100, *v*/*v*) to yield ten subfractions (Fr. D-1-Fr. D-10). Fraction C (30 g) was chromatographed over silica gel using a petroleum ether-ethyl acetate gradient (100:0 to 0:100, *v*/*v*) to afford ten subfractions (Fr. C-1-Fr. C-10). Fraction Fr. D-3/4 (14.7 g) was recrystallized from methanol or ethanol to yield compounds **2** (10 mg), **3** (4 mg), and **4** (6 mg). Fraction Fr. D-6/7 (3.5 g) was further purified by semipreparative HPLC (Venusil, 21.2 mm × 250 mm, 5 μm; methanol-water, 60:40, *v*/*v*; flow rate 8 mL/min) to afford compounds **5** (80 mg), **6** (800 mg), and **7** (162 mg). Fractions Fr. D-9 (1.5 g) and Fr. C-10 (1.5 g) were combined and further purified by silica gel column chromatography (dichloromethane-methanol), Sephadex LH-20 chromatography (dichloromethane-methanol, 1:1, *v*/*v*), and semipreparative HPLC (65% methanol) to yield compounds **8** (27 mg) and **9** (10 mg). A schematic separation procedure is shown in Figure S1.

### 4.3. Antioxidant Assays

#### 4.3.1. DPPH• Scavenging Assay

The DPPH• scavenging activity was evaluated according to the published protocol with modifications [41]. Briefly, 100 μL of each test sample (serially diluted in methanol) was measured with 100 μL freshly prepared DPPH• solution (0.1 mmol/L in methanol) in 96-well plates. After dark-incubation at 25 °C for 30 min, absorbance was measured at 517 nm using a microplate reader (SpectraMax iD5, Molecular Devices, San Jose, CA, USA). Vitamin C (0.001–0.5 mg/mL) served as the positive control. Each sample was done in triplicate, and results were expressed as *IC*_50_ values calculated from dose–response curves.

DPPH• scavenging activity was calculated according to the following Equation (1):

(1) $$\mathrm{DPPH}\;\mathrm{radical}\;\mathrm{scavenging}\;\mathrm{rate}(\%)\;=\;[1\;-\;\frac{A_{1}-{\;A}_{2}}{A_{3}}]\;\times \;100\%$$

where A_1_ was the absorbance of the tested sample group; A_2_ was the absorbance of the blank group (without DPPH); A_3_ was the absorbance of the control group (without sample).

#### 4.3.2. ABTS•+ Scavenging Assay

The ABTS•+ scavenging capacity was determined according to the established method with modifications [41]. Briefly, ABTS stock solution (7 mmol/L) was mixed with potassium persulfate solution (2.45 mmol/L) at an equal volume ratio and kept in the dark at 25 °C for 12–16 h to generate the ABTS radical cation solution. Before use, the resulting solution was diluted with methanol to an absorbance of 0.70 ± 0.02 at 734 nm. For the assay, 200 μL of sample solution at different concentrations in methanol was mixed with 200 μL of the ABTS working solution in a 96-well plate. After incubation in the dark at 25 °C for 30 min, the absorbance was measured at 734 nm using a SpectraMax iD5 microplate reader. Vitamin C (0.001–0.5 mg/mL) was used as the positive control. The ABTS radical-scavenging activity was calculated according to Equation (2), and the *IC*_50_ values were obtained from concentration-response curves.

ABTS•+ scavenging capacity was determined according to Equation (2):

(2) $$\mathrm{ABTS}\;\mathrm{radical}\;\mathrm{scavenging}\;\mathrm{activity}(\%)\;=\;[1\;-\;\frac{A_{1}\;-\;A_{2}}{A_{3}}]\;\times \;100\%$$

where A_1_ was the absorbance of the tested sample group; A_2_ was the absorbance of the blank group; A_3_ was the absorbance of the control group (without sample).

#### 4.3.3. FRAP Assay

The FRAP assay was performed according to the optimized protocol [42]. Briefly, The FRAP solution was prepared by mixing 300 mmol/L acetate buffers (pH 3.6), 10 mmol/L TPTZ (2,4,6-tris(2-pyridyl)-s-triazine) in 40 mmol/L HCl, and 20 mmol/L FeCl_3_•6H_2_O at a 10:1:1 volume ratio. The mixture was activated at 37 °C for 10 min. Afterward, 20 μL test sample (methanolic solution) was combined with 180 μL FRAP reagent to form a 200 μL reaction system. After 10 min incubation at 37 °C in the dark, absorbance was measured at 593 nm using a microplate reader (SpectraMax iD5). Vitamin C (0.001–0.5 mg/mL) served as the positive control. The reaction for each sample was done in triplicate. Results were expressed as the *EC*_50_ value.

### 4.4. HPLC Analysis

#### 4.4.1. Standard Solution Preparation

Authentic standards of pinoresinol (catalog no. DST200905-150, Chengdu Desite Biotechnology Co., Ltd., Chengdu, China), epipinoresinol (catalog no. DST180923-140, Chengdu Desite Biotechnology Co., Ltd., Chengdu, China), pinoresinol monomethyl ether (catalog no. BBP07378, Yunnan Xili Biotechnology Co., Ltd., Kunming, China) and phillygenin (catalog no. MUST-20060710, Chengdu Must Bio-Technology Co., Ltd., Chengdu, China) (10.00 mg each) were dissolved in HPLC-grade methanol and volumetrically adjusted to 10 mL in amber volumetric flasks, yielding stock solutions of 1.0 mg/mL. Working standards (0.02–0.5 mg/mL) were prepared by serial dilution for calibration curves.

#### 4.4.2. Sample Solution Preparation

FSLBT powder (0.5 g) was accurately weighed into a 50 mL conical tube, mixed with 15 mL 70% methanol (*v*/*v*), and ultrasonicated at 25 °C for 30 min. After cooling, the extract was adjusted to original weight with methanol to compensate for solvent evaporation, then centrifuged. The supernatant was filtered through 0.22 μm nylon membranes (Millipore, Bedford, MA, USA) prior to injection.

#### 4.4.3. HPLC Chromatographic Conditions

Chromatographic analysis was performed using an Agilent 1260 HPLC system (Agilent Technologies, Santa Clara, CA, USA) equipped with a Venusil XBP C18 column (4.6 mm × 250 mm, 5 μm; Agela Technologies). The mobile phase consisted of: A: 0.3% (*v*/*v*) glacial acetic acid in ultrapure water (pH 2.8); B: Acetonitrile (HPLC grade, Fisher, Waltham, MA, USA). The gradient elution (0~25 min: 33–45% B). The flow rate was 1.0 mL/min, the column temperature was 25 °C, the injection volume was 10 μL, and the detection wavelength was 280 nm. The quantification of pinoresinol, epipinoresinol, pinoresinol monomethyl ether, and phillygenin was performed using external calibration curves (0.02–0.5 mg/mL, R^2^ > 0.999). Each sample was tested in triplicates.

#### 4.4.4. Quantification of Lignan Aglycones in FSLBT by HPLC

The four major lignan aglycones (phillygenin, epipinoresinol, pinoresinol, and pinoresinol monomethyl ether) were quantified using HPLC-UV at 280 nm. Calibration curves were established using the corresponding reference standards with linear regression of peak area (y) versus concentration (x, mg/mL). Each FSLBT sample (*n* = 3 batches) was analyzed in triplicate.

The percentage content of each compound in FSLBT (%, *w*/*w*) was calculated as the mass of the compound determined from the HPLC peak area, divided by the mass of FSLBT powder used for extraction, and multiplied by 100. The mean ± SD of three independent batches is reported.

### 4.5. Statistical Analysis

Statistical analyses were performed using GraphPad Prism 8.0.2 (GraphPad Software). All data were expressed as mean ± standard deviation (SD) from three independent experiments.

## 5. Conclusions

Nine compounds were isolated in the FSLBT, including five furofuran lignans (**1**, **5**–**7**, **9**), and four pentacyclic triterpenic acids (**2**–**4**, **8**). Among these compounds **5** and **6** were isolated from FSLBT but not previously reported in FSL, indicating that fermentation is associated with alterations in the lignan profile. Phillygenin, pinoresinol monomethyl ether, pinoresinol, and epipinoresinol had antioxidant activity in DPPH•, ABTS•+ and FRAP assays, with epipinoresinol and pinoresinol showing the highest potency due to their dual ortho-methoxy-hydroxyl pairs. Quantitative HPLC analysis showed phillygenin as the most abundant lignan (~4%), whose high concentration may compensate for its moderate activity to play a dominant role in FSLBT’s overall antioxidant effects. These results enrich our knowledge of the bioactive components of FSLBT and provide a phytochemical basis for further investigation of its antioxidant properties.

## Figures and Tables

**Table 1:** ^1^H and ^13^C NMR Spectral Data of Lignan Compounds **1**, **5**, **6**, **7** ^a^.

NO.	1		5		6		7	
	*δ*_H_	*δ*_C_	*δ*_H_	*δ*_C_	*δ*_H_	*δ*_C_	*δ*_H_	*δ*_C_
1		133.2		133.9		133.6	3.03 m	132.0
2	7.26 s	109.3	6.89 d (1.6)	110.9	6.93 d (1.6)	109.3	4.87 d (4.5)	107.7
3		148.2		149.1		146.7		149.1
4		145.5		146.7		145.3	3.87 dd (8.7 3.4)	147.3
5	6.86 s	114.4	6.77 d (12)	119.4	6.87 d (8)	14.3	3.03 m	116.1
6	6.94 s	119.3	6.75 dd (1.6 12)	120.1	6.89 dd (8.2 1.7)	18.2	4.87 d (4.5)	119.9
7	4.44 d (7.1)	87.8		89.4	4.78 d (4.3)	85.9		84.9
8	2.92 m	54.6	3.26 m	55.6	3.13 m	54.1	3.87 dd (8.7 3.4)	54.2
9	4.14 s	69.8	4.36 d (9.6)	72.0	4.28 m	71.7		71.2
1′		131.1		131.3		132.9		132.0
2′		108.7		110.6	6.92 s	108.7	6.98 m	107.7
3′		149.0		148.8		149.2		149.1
4′		146.9		147.4		148.7	4.38 dd (8.7 6.9)	147.3
5′		111.3		116.0	6.91 m	111.2	6.98 m	116.1
6′		117.9		116.1	6.91 m	118.9	6.84 dd (8.0 2.0)	119.9
7′	4.87 d (5.5)	82.2		83.5	4.77 d (4.3)	85.7		84.9
8′	3.31 m	50.3		51.3	3.13 m	54.1	4.38 dd (8.7 6.9)	54.2
9′	3.85 s	71.2		70.6	4.28 m	71.7		71.2
CH_3_O-3	3.91 s	56.1	3.798 s	56.5	3.93 s	55.9	3.89 s	56.4
CH_3_O-3′	3.90 s		3.804 s		3.92 s		3.89 s	
CH_3_O-4						55.9		
CH_3_O-4′	3.88 s	56.1		56.5	3.90 s			

^a^ NMR data were measured in CDCl_3_ (Compounds **1** and **6**) and CD_3_OD (Compounds **5** and **7**) at 600 MHz for ^1^H-NMR and at 150 MHz for ^13^C-NMR. Proton coupling constants (*J*) in Hz are given in parentheses.

**Table 2:** Antioxidant activities of the four lignan compounds by DPPH•, ABTS•+, and FRAP methods.

Compounds	*IC*_50_ (μg/mL)		*EC*_50_ (mg/mL)
	DPPH•	ABTS•+	FRAP
Phillygenin	43.26 ± 4.46	54.27 ± 0.36	1.63 ± 0.02
Pinoresinol monomethyl ether	46.40 ± 1.22	72.71 ± 0.58	1.90 ± 0.07
Epipinoresinol	20.32 ± 1.34	37.29 ± 1.05	1.53 ± 0.01
Pinoresinol	20.98 ± 0.73	42.07 ± 0.55	1.58 ± 0.03
Vitamin C	12.67 ± 0.27	36.51 ± 0.44	1.31 ± 0.07

Note: Values are mean ± standard deviations from three experiments (*n* = 3).

**Table 3:** Quantification of the four major lignan aglycones in FSLBT (%) ^a^.

Lot No.	Pinoresinol	Epipinoresinol	Pinoresinol Monomethyl Ether	Phillygenin
S1	0.63 ± 0.006	0.48 ± 0.001	0.26 ± 0.001	4.73 ± 0.008
S2	0.32 ± 0.011	0.20 ± 0.001	0.14 ± 0.002	3.14 ± 0.024
S3	0.22 ± 0.003	0.09 ± 0.001	0.13 ± 0.003	2.56 ± 0.052
Mean ± SD	0.39 ± 0.21	0.26 ± 0.20	0.18 ± 0.07	3.48 ± 1.12

^a^ Percentages (%, *w*/*w*) were calculated as the mass of compound per mass of FSLBT powder. Values are presented as mean ± SD of three independent batches (*n* = 3).

## Data Availability

The data are included within the article and Supplementary Materials.

## Supplementary Materials

The following supporting information can be downloaded at: https://www.mdpi.com/article/10.3390/molecules31101687/s1.

## Abbreviations

ABTS	2:2′-Azino-bis(3-ethylbenzothiazoline-6-sulfonic acid)
DMSO	Dimethyl sulfoxide
DPPH	2,2-Diphenyl-1-picrylhydrazyl
*EC*_50_	Half maximal effective concentration
EP	Epipinoresinol
FRAP	Ferric reducing antioxidant power
FSL	*Forsythia suspensa* leaves
FSLBT	*Forsythia suspensa* leaves black tea
FSLGT	*Forsythia suspensa* leaves green tea
FSLT	*Forsythia suspensa* leaves tea
HPLC	High-performance liquid chromatography
HRESIMS	High-resolution electrospray ionization mass spectrometry
*IC*_50_	Half maximal inhibitory concentration
NMR	nuclear magnetic resonance
PME	Pinoresinol monomethyl ether
PR	Pinoresinol
PY	Phillygenin
ROS	Reactive oxygen species
TCM	Traditional Chinese medicine
Vc	Vitamin C

## References

[1] Wang Z., Xia Q., Liu X., Liu W., Huang W., Mei X., Luo J., Shan M., Lin R., Zou D. (2018). Phytochemistry, pharmacology, quality control and future research of *Forsythia suspensa* (Thunb.) Vahl: A review. J. Ethnopharmacol..

[2] Xiang K.L., Liu R.X., Zhao L., Xie Z.P., Zhang S.M., Dai S.J. (2020). Labdane diterpenoids from *Forsythia suspensa* with anti-inflammatory and anti-viral activities. Phytochemistry.

[3] Cho H.E., Ahn S.Y., Son I.S., Hwang G.H., Kim S.C., Woo M.H., Lee S.H., Son J.K., Hong J.T., Moon D.C. (2011). HPLC-tandem mass spectrometric analysis of the marker compounds in Forsythiae Fructus and multivariate analysis. Nat. Prod. Sci..

[4] Li S.F., Zhang L.W., Zhan Z.L. (2022). Herbal textual research on Forsythiae Fructus in famous classical formulas. Chin. J. Exp. Tradit. Med. Formulae.

[5] Ge Y., Wang Y., Chen P., Wang Y., Hou C., Wu Y., Zhang M., Li L., Huo C., Shi Q. (2016). Polyhydroxytriterpenoids and phenolic constituents from *Forsythia suspensa* (Thunb.) Vahl leaves. J. Agric. Food Chem..

[6] Zhang Q., Jia C., Xu H.Y., Wang Y.F., Zhang M.L., Huo C.C., Shi Q., Yu S.H. (2012). Chemical constituents of plants from the genus Forsythia. Mini-Rev. Org. Chem..

[7] Hou G.X., Yang J.X. (2010). Effects of extracts from *Forsythia suspensa* leaves on modulating blood lipids and protecting liver of hyperlipidemic mice. J. Henan Univ. Nat. Sci..

[8] Michalak B., Filipek A., Chomicki P., Pyza M., Woźniak M., Żyżyńska-Granica B., Piwowarski J.P., Kicel A., Olszewska M.A., Kiss A.K. (2018). Lignans from *Forsythia × intermedia* leaves and flowers attenuate the pro-inflammatory function of leukocytes and their interaction with endothelial cells. Front. Pharmacol..

[9] Kang W., Wang J. (2010). In vitro antioxidant properties and in vivo lowering blood lipid of *Forsythia suspensa* leaves. Med. Chem. Res..

[10] Jiao J., Gai Q., Luo M., Wang W., Gu C., Zhao C., Zu Y., Wei F., Fu Y. (2013). Comparison of main bioactive compounds in tea infusions with different seasonal *Forsythia suspensa* leaves by liquid chromatography-tandem mass spectrometry and evaluation of antioxidant activity. Food Res. Int..

[11] Yuan J., Liu X., Yang J., Cui X. (2014). *Forsythia suspensa* leaves, a plant resource: Extraction, purification and antioxidant activity of main active compounds. Eur. Food Res. Technol..

[12] Kang W.Y., Wang J.M., Zhang L. (2010). α-Glucosidase inhibitors from leaves of *Forsythia suspensa* in Henan Province. Zhongguo Zhongyao Zazhi.

[13] Gui L., Wang S., Wang J., Liao W., Chen Z., Pan D., Xia H., Sun G., Tian S. (2024). Effects of forsythin extract in Forsythia leaves on intestinal microbiota and short-chain fatty acids in rats fed a high-fat diet. Food Sci. Hum. Wellness.

[14] Guo J., Tang J.K., Wang B.F., Yan W.R., Li T., Guo X.J., Zhang L., Wang T., Sun Q.Y., Zhang L.W. (2022). Phillygenin from *Forsythia suspensa* leaves exhibits analgesic potential and anti-inflammatory activity in carrageenan-induced paw edema in mice. J. Food Biochem..

[15] Liang Z., Huang Y., Zhang P., Fang Z. (2023). Impact of fermentation on the structure and antioxidant activity of selective phenolic compounds. Food Biosci..

[16] Yang F., Chen C., Ni D., Yang Y., Tian J., Li Y., Chen S., Ye X., Wang L. (2023). Effects of fermentation on bioactivity and the composition of polyphenols contained in polyphenol-rich foods: A review. Foods.

[17] Wang D.H., Wang M.Y., Shen W.H., Yuan J.F. (2021). Analysis of chemical compounds and toxicological evaluation of *Forsythia suspensa* leaves tea. Food Sci. Biotechnol..

[18] Kitagawa S., Hisada S., Nishibe S. (1984). Phenolic compounds from Forsythia leaves. Phytochemistry.

[19] Bringmann G., Saeb W., Assi L.A., François G., Narayanan A.S.S., Peters K., Peters E.M. (1997). Betulinic acid: Isolation from *Triphyophyllum peltatum* and *Ancistrocladus heyneanus*, antimalarial activity, and crystal structure of the benzyl ester. Planta Med..

[20] Mahato S.B., Kundu A.P. (1994). 13C NMR spectra of pentacyclic triterpenoids: A compilation and some salient features. Phytochemistry.

[21] Chandramu C., Manohar R.D., Krupadanam D.G.L., Dashavantha R.V. (2003). Isolation, characterization and biological activity of betulinic acid and ursolic acid from *Vitex negundo* L.. Phytother. Res..

[22] Rahman M.M.A., Dewick P.M., Jackson D.E., Lucas J.A. (1990). Lignans of Forsythia intermedia. Phytochemistry.

[23] Kitagawa S., Nishibe S., Benecke R., Thieme H. (1988). Phenolic compounds from Forsythia leaves. II. Chem. Pharm. Bull..

[24] Yamauchi S., Ina T., Kirikihira T., Masuda T. (2004). Synthesis and antioxidant activity of oxygenated furofuran lignans. Biosci. Biotechnol. Biochem..

[25] Rana A., Samtiya M., Dhewa T., Mishra V., Aluko R.E. (2022). Health benefits of polyphenols: A concise review. J. Food Biochem..

[26] Tsiftsoglou O.S., Stefanakis M.K., Kalpourtzi E.N., Hadjipavlou-Litina D.I., Lazari D.M. (2022). Chemical constituents isolated from the aerial parts of *Helleborus cyclophyllus* (A. Braun) Boiss. (Ranunculaceae), evaluation of their antioxidant and anti-inflammatory activity in vitro and virtual screening of molecular properties and bioactivity score. Nat. Prod. Res..

[27] Dimitriadis K.M., Karavergou S., Tsiftsoglou O.S., Karapatzak E., Paschalidis K., Hadjipavlou-Litina D., Charalambous D., Krigas N., Lazari D. (2024). Nutritional value, major chemical compounds, and biological activities of *Petromarula pinnata* (Campanulaceae)—A unique nutraceutical wild edible green of Crete (Greece). Horticulturae.

[28] Chafouz R., Karavergou S., Tsiftsoglou O.S., Maskovic P., Lazari D. (2024). *Ganoderma adspersum* (Ganodermataceae): Investigation of its secondary metabolites and the antioxidant, antimicrobial, and cytotoxic potential of its extracts. Int. J. Mol. Sci..

[29] Tsiftsoglou O., Lazari D. (2018). Chemical constituents isolated from the rhizomes of *Helleborus odorus* subsp. cyclophyllus (Ranunculaceae). Biochem. Syst. Ecol..

[30] Charlton N.C., Mastyugin M., Török B., Török M. (2023). Structural features of small molecule antioxidants and strategic modifications to improve potential bioactivity. Molecules.

[31] Ponomarenko J., Dizhbite T., Lauberts M., Viksna A., Dobele G., Bikovens O., Telysheva G. (2014). Characterization of softwood and hardwood LignoBoost kraft lignins with emphasis on their antioxidant activity. BioResources.

[32] Zhu M.Z., Li N., Zhou F., Ouyang J., Lu D.M., Xu W., Li J., Lin H.Y., Zhang Z., Xiao J.B. (2020). Microbial bioconversion of the chemical components in dark tea. Food Chem..

[33] Wang Q.B., Zhang J.S., Yan Z., Wang L.Z., Zhang L.W. (2020). Research on fermentation technology for the conversion from phillyrin to phillygenin in *Forsythia suspensa* leaves. Chem. Res. Appl..

[34] Gao S.M., Yan R.P., Liu Z.Q., Duan Y., Zhang L.W. (2023). Study on lignan aglycones production from lignan glycosides transformed by endogenous enzymes in *Forsythia suspensa* leaves. Chem. Res. Appl..

[35] Ma L.J., Liu J.Y., Fu W.Y., Bi H.C., Yuan D., Wan J.B. (2025). β-Glucosidase mediates ginsenoside hydrolysis in the leaves of Panax species. Ind. Crops Prod..

[36] Ji Y.L., Feng X., Chang Y.Q., Zheng Y.G., Hou F.J., Zhang D., Guo L. (2024). Chemical characterization of different parts of *Forsythia suspensa* and α-glucosidase and pancreatic lipase inhibitors screening based on UPLC-QTOF-MS/MS and plant metabolomics analysis. Arab. J. Chem..

[37] Osama S., El Sherei M., Al-Mahdy D.A., Bishr M., Salama O. (2019). Effect of salicylic acid foliar spraying on growth parameters, γ-pyrones, phenolic content and radical scavenging activity of drought-stressed *Ammi visnaga* L. plant. Ind. Crops Prod..

[38] Boulebd H., Zine Y., Khodja I.A., Mermer A., Demir A., Debache A. (2022). Synthesis and radical scavenging activity of new phenolic hydrazone/hydrazide derivatives: Experimental and theoretical studies. J. Mol. Struct..

[39] Wang C., Wu R., Zhang S., Gong L., Fu K., Yao C., Peng C., Li Y. (2023). A comprehensive review on pharmacological, toxicity, and pharmacokinetic properties of phillygenin: Current landscape and future perspectives. Biomed. Pharmacother..

[40] Li J., Qin Q., Zha S.-H., Zhao Q.-S., Li H., Liu L.-P., Hou S.-B., Zhao B. (2022). Green extraction of Forsythoside A, Phillyrin and Phillygenol from *Forsythia suspensa* leaves using a β-cyclodextrin-assisted method. Molecules.

[41] Tabakam G.T., Njoya E.M., Chukwuma C.I., Mashele S.S., Nguekeu Y.M.M., Tene M., Awouafack M.D., Makhafola T.J. (2024). The antioxidant and anti-inflammatory activities of the methanolic extract, fractions, and isolated compounds from *Eriosema montanum* Baker f. (Fabaceae). Molecules.

[42] Cañas S., Rebollo-Hernanz M., Bermúdez-Gómez P., Rodríguez-Rodríguez P., Braojos C., Gil-Ramírez A., Benítez V., Aguilera Y., Martín-Cabrejas M.A. (2023). Radical scavenging and cellular antioxidant activity of the cocoa shell phenolic compounds. Antioxidants.

