# Supplementary material for: Chemical Compounds and Antioxidant Activity of Forsythia suspensa Leaves Black Tea
Source: Molecules. 2026 May 16;31(10):1687. doi: 10.3390/molecules31101687 (PMC13210340; doi:10.3390/molecules31101687)
Supplement: Supplementary file 1 [file molecules-31-01687-s001.zip › molecules-4296896-supplementary.pdf]

Supporting Information

Chemical Compounds and Antioxidant Activity of *Forsythia suspensa* Leaves Black Tea

Shuheng Wang [1,2], Qi Du [1], Junwen Ma [1,2], Xin Yuan [1], Shifei Li [1], Xiaoxia Gao [2,3*], Liwei Zhang [1*]

1 Key Laboratory of Chemical Biology and Molecular Engineering of Ministry of Education, Institute of Molecular Science, Shanxi University, No. 92, Wucheng Road, Taiyuan 030006, China; 202214301003@email.sxu.edu.cn (S.W.)

2 Modern Research Center for Traditional Chinese Medicine, Shanxi University, No. 92, Wucheng Road, Taiyuan 030006, China

3 Key Laboratory of Research and Utilization of Bioactive Components in Famous Shanxi Medicinal Materials, Shanxi University, No. 92, Wucheng Road, Taiyuan 030006, China

* Correspondence: gaoxiaoxia@sxu.edu.cn (X.G.); lwzhang@sxu.edu.cn (L.Z.)

1. Isolation scheme

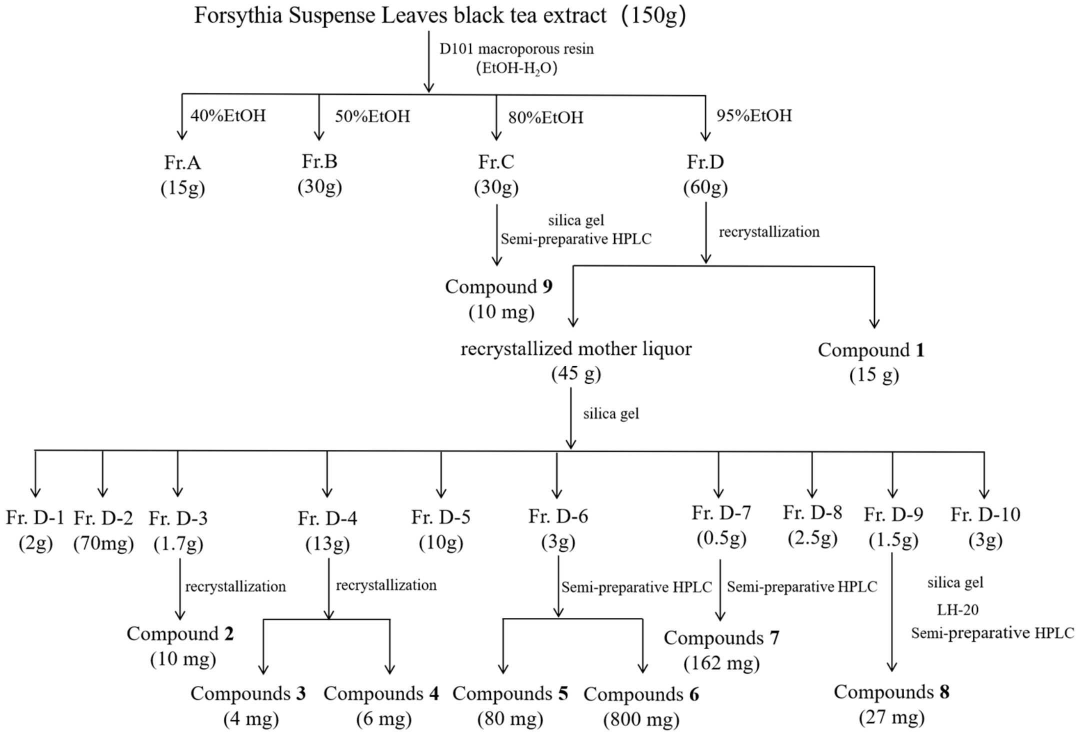

Figure S1. Methodology of extraction and isolation of compounds from FSLBT

2. HRESIMS Data for All Isolated Compounds (**1–9**)

Phillygenin (1): white crystals; HRESIMS *m/z* 395.14655 $[M+Na]^+$, 371.15045 $[M-H]^-$ (calcd for $C_{21}H_{24}O_6$, 372.41200); **Betulinic acid (2)**: white crystals; HRESIMS *m/z* 457.36767 $[M+H]^+$; 455.35385 $[M-H]^-$ (calcd for $C_{30}H_{48}O_3$, 456.36034); **Oleanolic acid (3)**: white acicular crystals; HRESIMS *m/z* 479.34967 $[M+Na]^+$; 455.35355 $[M-H]^-$ (calcd for $C_{30}H_{48}O_3$, 456.36035); **Ursolic acid (4)**: white powder; HRESIMS *m/z* 479.34964 $[M+Na]^+$; 455.35362 $[M-H]^-$ (calcd for $C_{30}H_{48}O_3$, 456.36029); **Epipinoresinol (5)**: white powder; HRESIMS *m/z* 381.13068 $[M+Na]^+$; 357.13495 $[M-H]^-$ (calcd for $C_{20}H_{22}O_6$, 358.38500); **Pinoresinol monomethyl ether (6)**: white powder; HRESIMS *m/z* 395.14661 $[M+Na]^+$; 371.15063 $[M-H]^-$ (calcd for $C_{21}H_{24}O_6$, 372.41166); **Pinoresinol (7)**: white powder; HRESIMS *m/z* 381.13088 $[M+Na]^+$; 357.13476 $[M-H]^-$ (calcd for $C_{20}H_{22}O_6$, 358.39001); **Esculentic acid (8)**: white powder; HRESIMS *m/z* 511.33936 $[M+Na]^+$; 487.34343 $[M-H]^-$ (calcd for $C_{30}H_{48}O_5$, 488.35017); **Phillyrin (9)**: white powder; HRESIMS *m/z* 557.19958 $[M+Na]^+$; 533.20365 $[M-H]^-$ (calcd for $C_{27}H_{34}O_{11}$, 534.21011).

3. NMR Data of Lignans and Pentacyclic Triterpenic Acids from FSLBT

Table S1. ^{1}H and ^{13}C NMR Spectral Data of Lignan Compound **9**[a.]

NO.	9		NO.	9	
	δ_H	δ_C		δ_H	δ_C
1		135.7	6′	6.87 brs	109.8
2	6.97 d(1.5)	118.6	7′	4.80 d(5.9)	81.7
3		149.3	8′	3.17 t(4.9)	81.7
4		146.3	9′	4.10 d(9.2)	70.1
5	7.05 d(8.4)	118.0	CH_3O-3	3.75 m	55.9
6	6.87 brs	115.6	CH_3O-3′	3.75 m	55.9
7	4.38 d(6.8)	87.1	CH_3O-4		
8	3.17 t(4.9)	56.1	CH_3O-4′	3.75 m	55.9
9	4.10 d(9.2)	70.7	1″	4.88 d(7.1)	100.5
1′		131.6	2″	3.39 m	73.6
2′	6.93 d(1.4)	110.8	3″	3.39 m	77.4
3′		148.9	4″	3.27 m	69.4
4′		148.0	5″	3.27 m	77.3
5′	6.92 d(5.2)	111.9	6″	3.66 m<br>3.45 dt (11.6 5.7)	61.1

[a] NMR data were measured in DMSO-d_6 (Compound **9**) at 600 MHz for ^{1}H-NMR and at 150 MHz for ^{13}C-NMR. Proton coupling constants (*J*) in Hz are given in parentheses.

Table S2. ^{1}H and ^{13}C NMR Spectral Data of Triterpenic Acid Compounds **2**, **3**, **4**, **8**[b.]

NO.	**2**		**3**		**4**		**8**	
	δ_H	δ_C	δ_H	δ_C	δ_H	δ_C	δ_H	δ_C
1		38.4		38.3		38.6		42.1
2		27.1		25.9		27.2	4.22 d (3.6)	65.2
3	4.26 m	76.7	3.24 dd (11.3 4.2)	79.0	3.24 dd (11.3 4.7)	79.0	3.71 d (3.6)	76.3
4		39.5		38.7		38.7		41.6
5		55.3		55.2		55.2	1.32 m	43.7
6		18.9		18.2		18.2		17.9
7		33.9		32.6		32.9		32.8
8		40.2		40.9		39.4		39.6
9		49.9		47.6		47.5		47.4
10		37.5		37.1		36.7		38.1
11		20.4		23.4		23.3		23.3
12		25.0	5.30 t (3.5)	122.6	5.28 t (3.6)	125.8	5.14 t (9.9)	124.9
13		38.2		143.5		137.9		138.8
14		41.9		41.5		42.0		42.2
15		30.1		27.6		28.0		28.0
16		31.7		22.9	2.04 td (13.6 4.7)	24.1		24.4
17		54.8		45.8		47.9		47.3
18		46.5		39.3	2.21 d (11.7)	52.6	2.12 d (16.8)	53.0
19	4.25 m	48.5		46.5		39.0		39.0
20		150.2		30.6		38.8		38.9
21		29.1		33.7		30.6		30.7
22		36.7		32.4		37.0		36.8
23		28.0	0.79 s	28.1	1.11 s	28.1	3.42 d (2.55)<br>3.20 d (2.55)	69.6
24	0.65 s	15.7	0.77 s	15.5	0.95 s	15.6	0.70 s	17.3
25	0.76 s	15.7	0.93 s	15.3	0.80 s	15.4	0.92 s	17.0
26	0,87 s	15.8	0.92 s	17.1	0.81 s	17.1	0.74 s	17.4
27	0.93 s	14.3	0.95 s	27.1	1.01 s	23.5	1.04 s	23.8
28		177.1		183.1		178.3		178.9
29	4.69 br<br>4.56 br	109.5	1.01 s	33.0	0.88 d (6.5)	21.1	0.82 d (9.6)	17.3
30	1.64 s	17.9	1.15 s	23.5	0.97 d (6.3)	17.0	0.91 d (9.6)	21.5

[b] NMR data were measured in DMSO-d_6 (Compounds **2** and **8**) and $CDCl_3$ (Compounds **3** and **4**) at 600 MHz for ^{1}H-NMR and at 150 MHz for ^{13}C-NMR. Proton coupling constants (*J*) in Hz are given in parentheses.

4. HPLC Calibration Curves of Phillygenin, Pinoresinol, Epipinoresinol, and Pinoresinol Monomethyl Ether

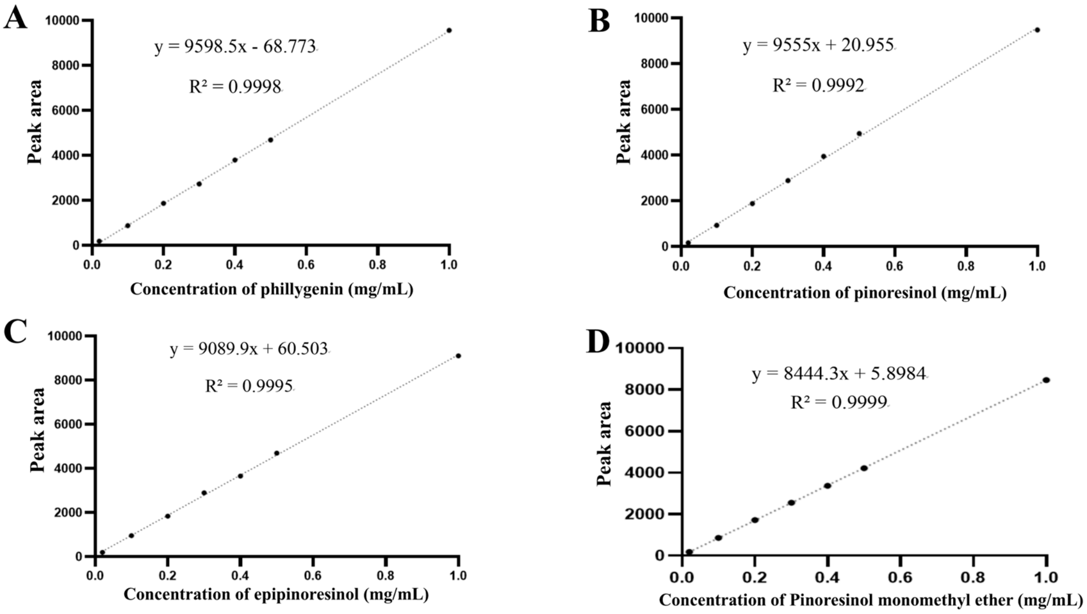

Figure S2. Calibration curves of four lignan aglycones used for quantitative HPLC analysis. (A) Phillygenin, (B) Pinoresinol, (C) Epipinoresinol, and (D) Pinoresinol monomethyl ether. The x-axis represents the concentration of each compound (mg/mL), and the y-axis represents the corresponding peak area (arbitrary units, AU). Linear regression equations and correlation coefficients (R^2) are indicated for each compound.

5. Method Validation for Quantification of Four Major Lignan Aglycones in FSLBT

Table S3. The results of precision experiment

samples	Phillygenin		Pinoresinol		Epipinoresinol		Pinoresinol monomethyl ether	
	Rt (min)	Peak Area	Rt (min)	Peak Area	Rt (min)	Peak Area	Rt (min)	Peak Area
1	21.803	1673.900	11.613	172.478	13.979	115.337	18.533	87.654
2	21.821	1680.523	11.641	174.095	13.971	114.194	18.540	86.135
3	21.828	1708.970	11.645	172.725	13.969	113.078	18.549	84.654
4	21.831	1704.373	11.650	178.499	13.946	122.703	18.550	83.320
5	21.808	1700.564	11.625	173.802	13.972	124.604	18.551	82.687
6	21.803	1698.357	11.634	176.528	13.965	116.376	18.554	87.014
RSD/%	0.058	0.825	0.118	1.349	0.081	2.966	0.043	2.365

Table S4. The results of repeatability experiment

samples	Phillygenin		Pinoresinol		Epipinoresinol		Pinoresinol monomethyl ether	
	Rt (min)	Peak Area	Rt (min)	Peak Area	Rt (min)	Peak Area	Rt (min)	Peak Area
1	21.794	1691.621	11.625	175.132	13.953	122.013	18.574	81.083
2	21.803	1691.154	11.634	174.451	13.960	124.744	18.564	85.324
3	21.815	1703.147	11.637	175.853	13.964	121.442	18.561	86.232
4	21.810	1697.021	11.635	172.453	13.966	123.864	18.537	84.811
5	21.809	1694.872	11.638	174.744	13.965	120.231	18.554	82.160
6	21.815	1697.643	11.643	174.310	13.972	123.487	18.551	85.156
RSD/%	0.037	0.262	0.051	0.654	0.046	1.378	0.068	2.408

Table S5. The results of stability experiment

Samples / Times	Phillygenin		Pinoresinol		Epipinoresinol		Pinoresinol monomethyl ether	
	Rt (min)	Peak Area	Rt (min)	Peak Area	Rt (min)	Peak Area	Rt (min)	Peak Area
0h	21.805	1704.021	11.635	179.121	13.971	118.212	18.564	83.475
2h	21.812	1684.421	11.642	173.742	13.946	121.784	18.537	83.694
4h	21.803	1687.198	11.628	172.382	13.965	123.312	18.551	86.415
6h	21.798	1685.512	11.632	175.045	13.953	122.075	18.533	84.321
8h	21.807	1688.714	11.622	172.484	13.964	128.769	18.549	82.985
10h	21.803	1683.520	11.635	175.451	13.965	121.785	18.551	84.612
12h	21.820	1693.142	11.640	173.785	13.969	120.235	18.561	83.671
RSD/%	0.033	0.424	0.059	1.326	0.065	2.679	0.061	1.339

Table S6. The results of sample recovery experiment (Phillygenin)

Sample Amount (mg)		Amount of Phillygenin in Sample (mg)	Amount of Phillygenin Spiked (mg)	Measured Phillygenin Amount (mg)	Recovery (%)	Mean Recovery (%)	RSD/%
	20.00	3.60	1.83	5.42	99.82		
50%	20.00	3.60	1.85	5.40	99.08	99.63	0.49
	20.00	3.60	1.76	5.36	100.00		
	20.00	3.60	3.66	7.25	99.86		
100%	20.00	3.60	3.59	7.23	100.56	100.14	0.37
	20.00	3.60	3.62	7.22	100.00		
	20.00	3.60	5.42	9.00	99.78		
150%	20.00	3.60	5.45	9.07	100.22	100.00	0.22
	20.00	3.60	5.33	8.93	100.00		

Table S7. The results of sample recovery experiment (Pinoresinol)

Sample Amount (mg)		Amount of Pinoresinol in Sample (mg)	Amount of Pinoresinol Spiked (mg)	Measured Pinoresinol Amount (mg)	Recovery (%)	Mean Recovery (%)	RSD/%
	20.00	0.33	0.18	0.52	101.96		
50%	20.00	0.33	0.15	0.49	102.08	101.35	1.15
	20.00	0.33	0.16	0.49	100.00		
	20.00	0.33	0.35	0.67	98.53		
100%	20.00	0.33	0.33	0.66	100.00	99.00	0.87
	20.00	0.33	0.33	0.65	98.48		
	20.00	0.33	0.50	0.84	101.20		
150%	20.00	0.33	0.48	0.83	102.47	101.22	1.22
	20.00	0.33	0.48	0.81	100.00		

Table S8. The results of sample recovery experiment (Epipinoresinol)

Sample Amount (mg)		Amount of Epipinoresinol in Sample (mg)	Amount of Epipinoresinol Spiked (mg)	Measured Epipinoresinol Amount (mg)	Recovery (%)	Mean Recovery (%)	RSD/%
	50.00	0.31	0.15	0.46	102.17		
50%	50.00	0.31	0.15	0.45	97.83	98.57	1.26
	50.00	0.31	0.16	0.46	97.87		
	50.00	0.31	0.31	0.63	101.61		
100%	50.00	0.31	0.32	0.62	98.41	100.01	1.60
	50.00	0.31	0.32	0.63	100.00		
	50.00	0.31	0.46	0.78	101.30		
150%	50.00	0.31	0.45	0.77	101.32	100.87	0.75
	50.00	0.31	0.46	0.77	100.00		

Table S9. The results of sample recovery experiment (Pinoresinol monomethyl ether)

Sample Amount (mg)		Amount of Pinoresinol monomethyl ether in Sample (mg)	Amount of Pinoresinol monomethyl ether Spiked (mg)	Measured Pinoresinol monomethyl ether Amount (mg)	Recovery (%)	Mean Recovery (%)	RSD/%
	50.00	0.50	0.25	0.76	101.33		
50%	50.00	0.50	0.24	0.75	101.35	100.89	0.77
	50.00	0.50	0.25	0.75	100.00		
	50.00	0.50	0.52	1.00	98.04		
100%	50.00	0.50	0.50	1.01	101.00	99.68	1.51
	50.00	0.50	0.50	1.00	100.00		
	50.00	0.50	0.99	1.51	101.34		
150%	50.00	0.50	0.99	1.50	100.67	100.45	1.02
	50.00	0.50	1.01	1.50	99.34		

6. ^{1}H and ^{13}C NMR spectral data of compounds **1-9** (600/150 MHz)

List of figures

Item	Subject
Figure. S3	^{1}H NMR of compound 1
Figure. S4	^{13}C NMR of compound 1
Figure. S5	^{1}H NMR of compound 2
Figure. S6	^{13}C NMR of compound 2
Figure. S7	^{1}H NMR of compound 3
Figure. S8	^{13}C NMR of compound 3
Figure. S9	^{1}H NMR of compound 4
Figure. S10	^{13}C NMR of compound 4
Figure. S11	^{1}H NMR of compound 5
Figure. S12	^{13}C NMR of compound 5
Figure. S13	^{1}H NMR of compound 6
Figure. S14	^{13}C NMR of compound 6
Figure. S15	^{1}H NMR of compound 7
Figure. S16	^{13}C NMR of compound 7
Figure. S17	^{1}H NMR of compound 8
Figure. S18	^{13}C NMR of compound 8
Figure. S19	^{1}H NMR of compound 9
Figure. S20	^{13}C NMR of compound 9

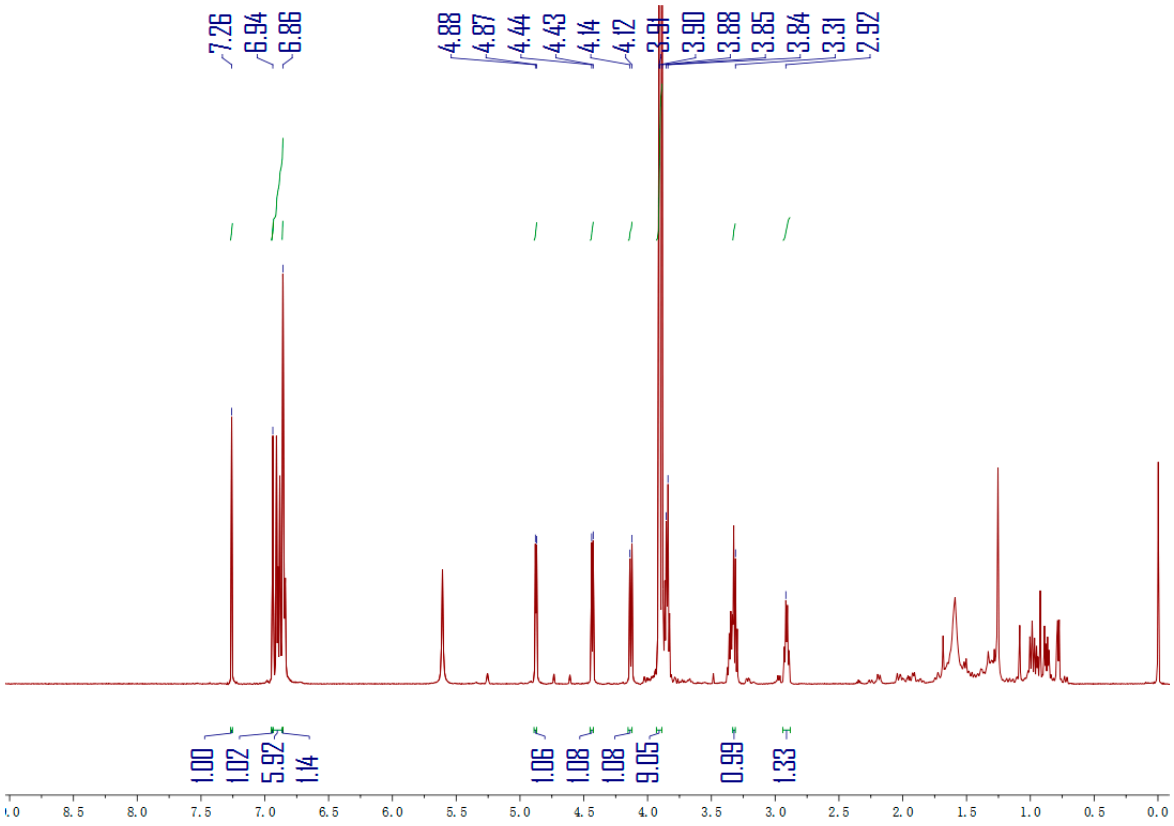

Figure S3. ^{1}H NMR of compound 1

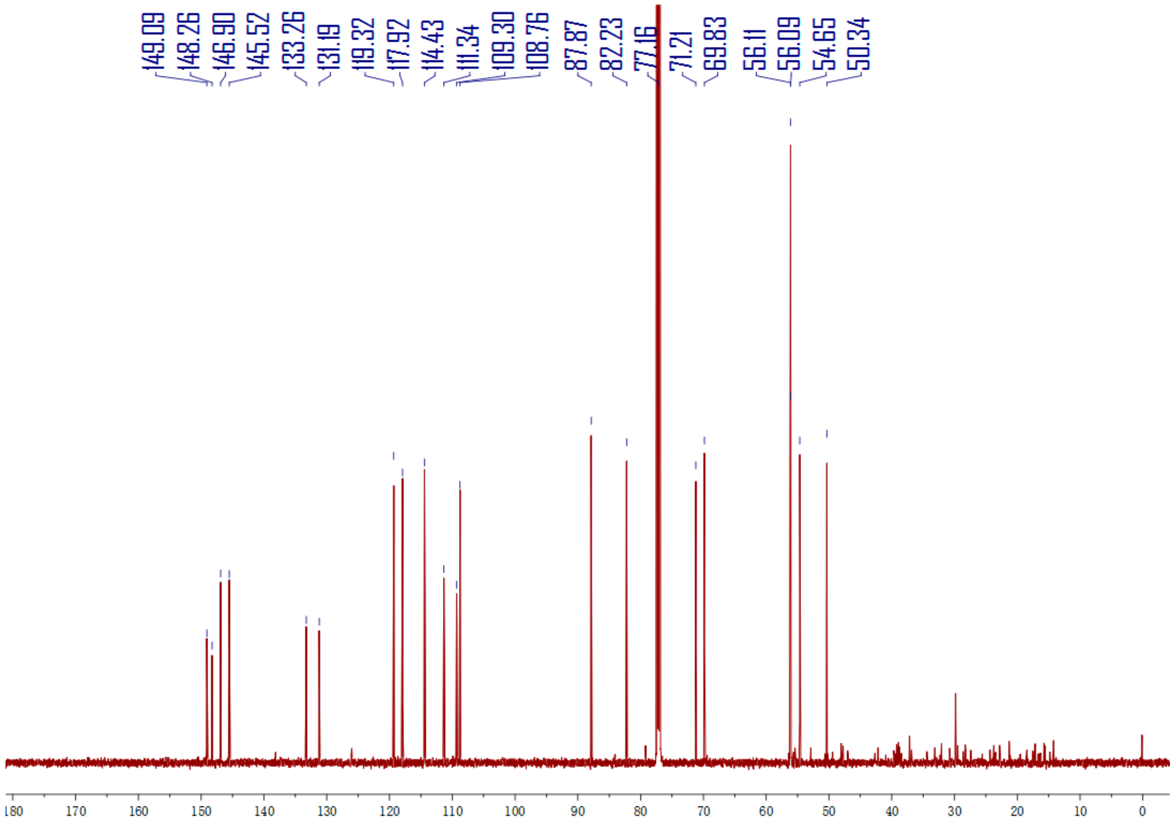

Figure S4. ^{13}C NMR of compound 1

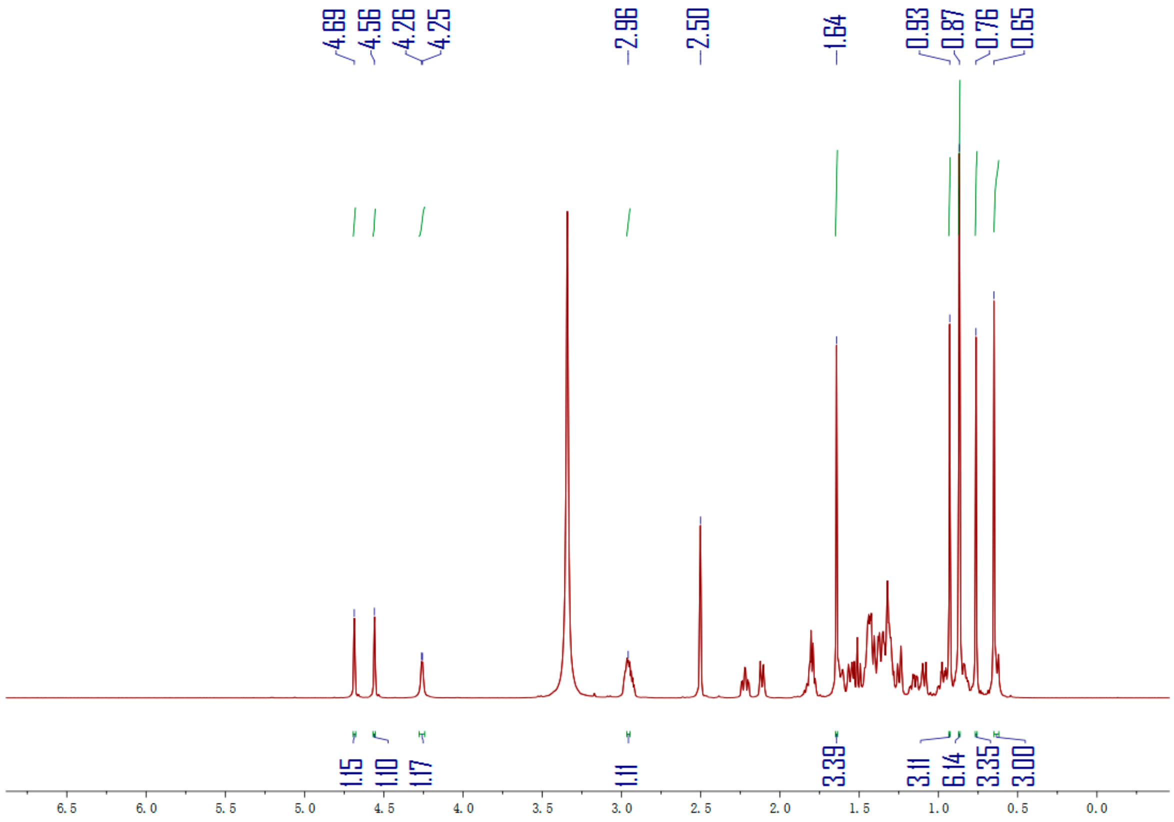

Figure S5. ^{1}H NMR of compound 2

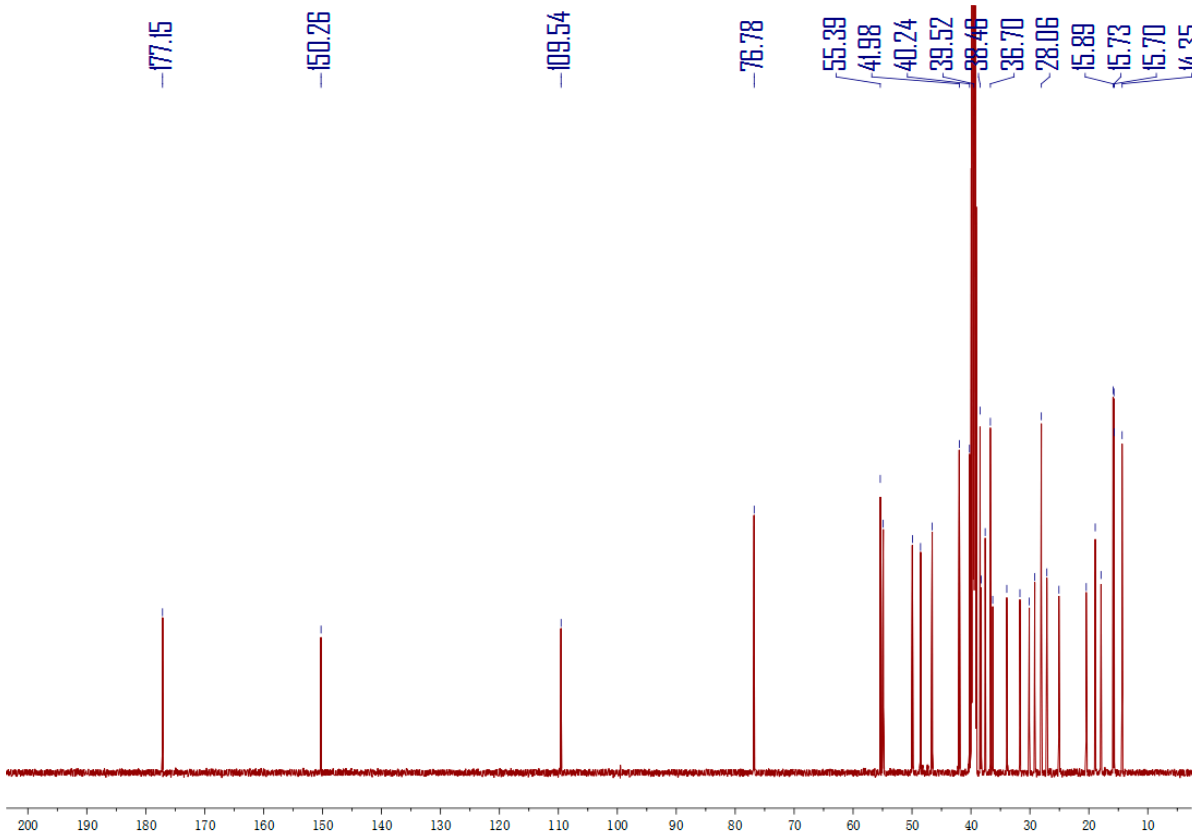

Figure S6. ^{13}C NMR of compound 2

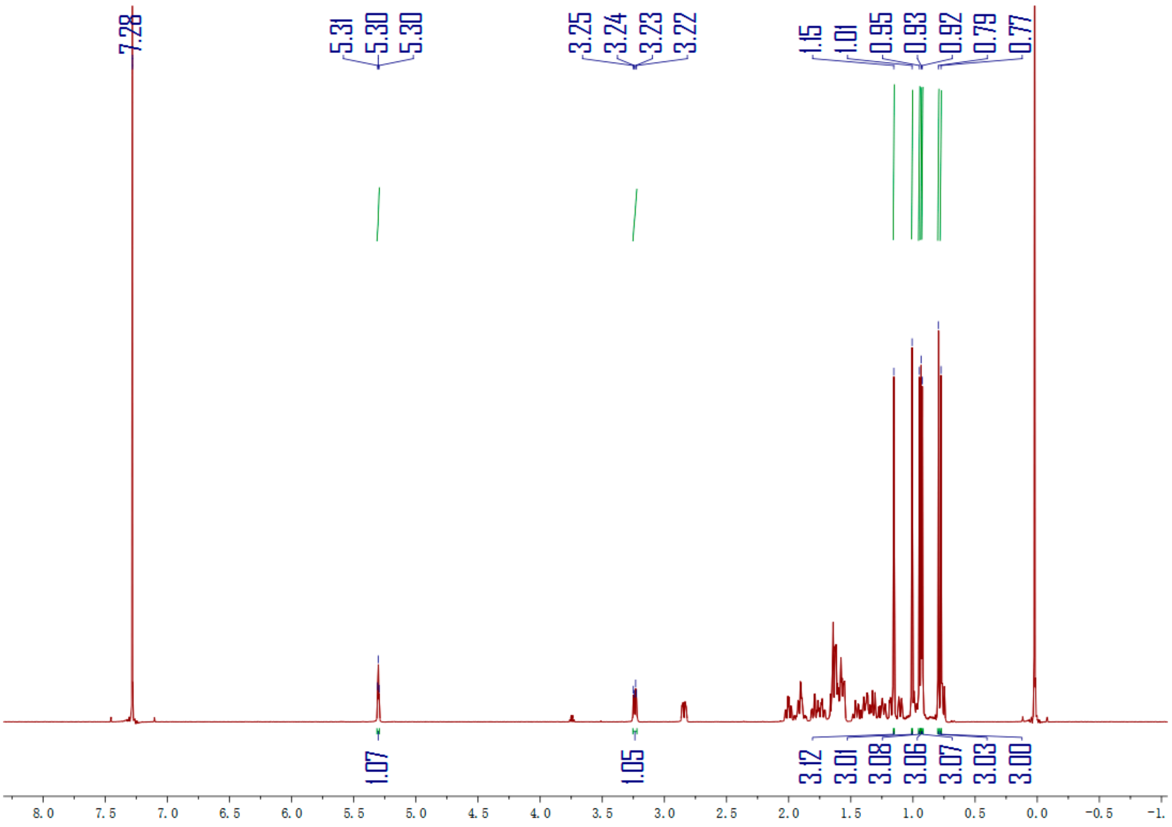

Figure S7. ^{1}H NMR of compound 3

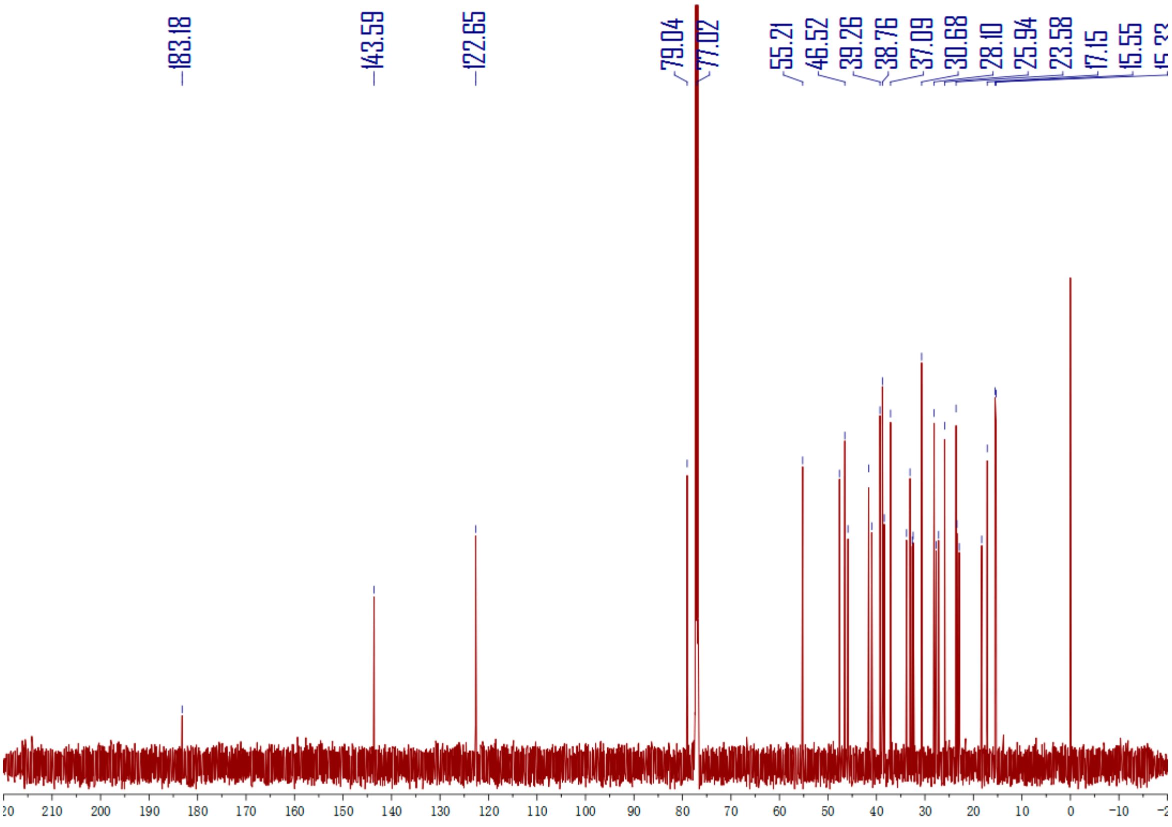

Figure S8. ^{13}C NMR of compound 3

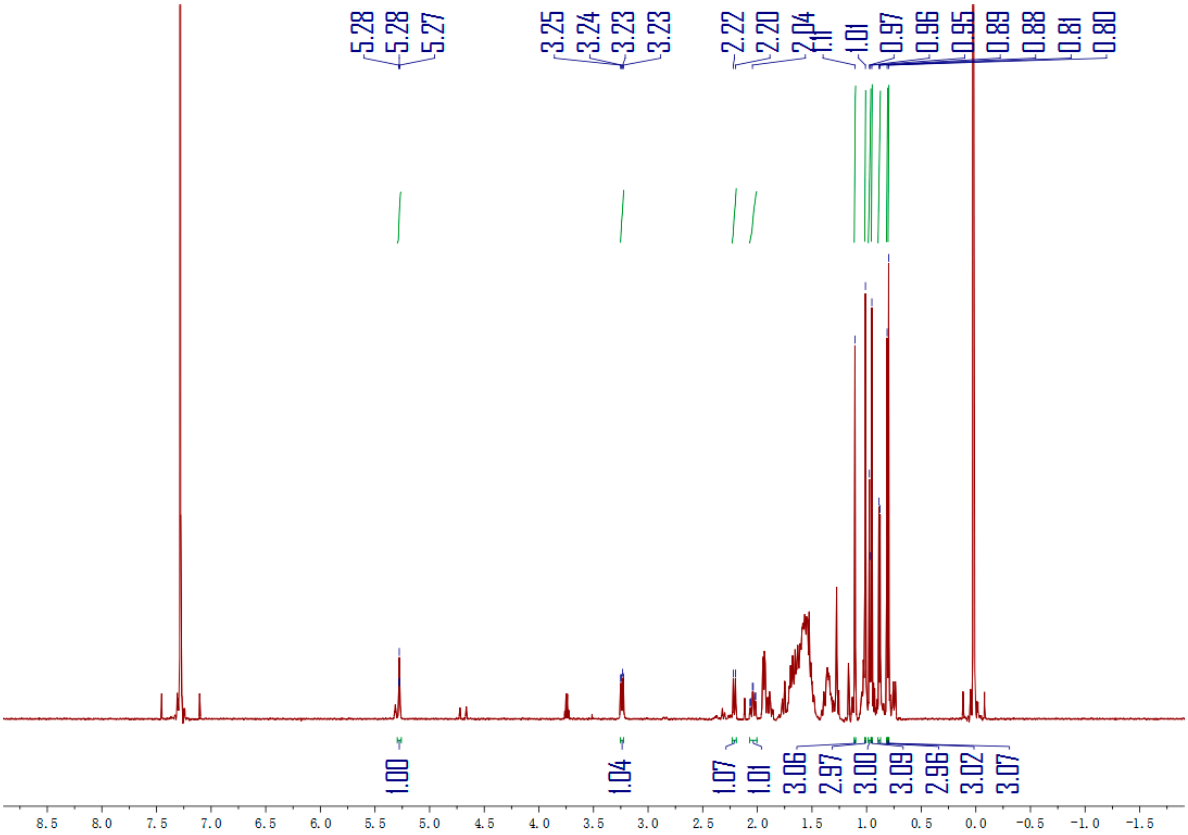

Figure S9. ^{1}H NMR of compound 4

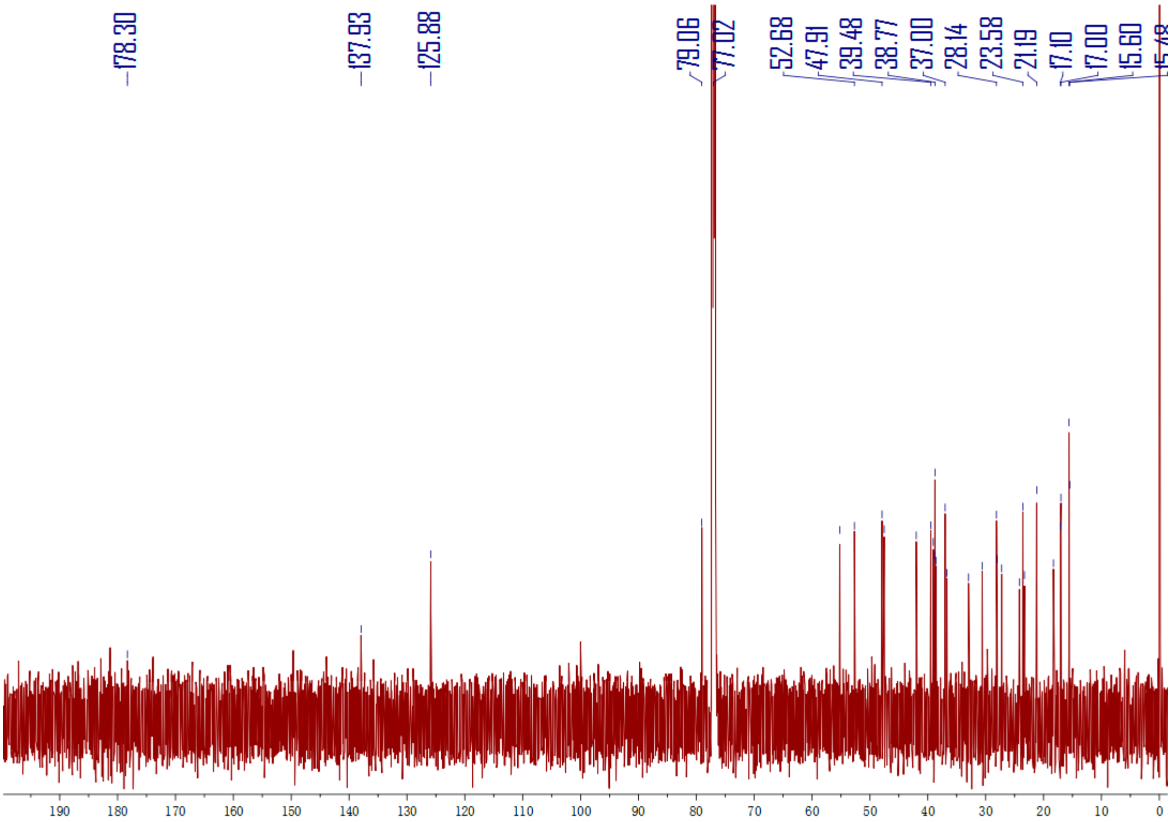

Figure S10. ^{13}C NMR of compound 4

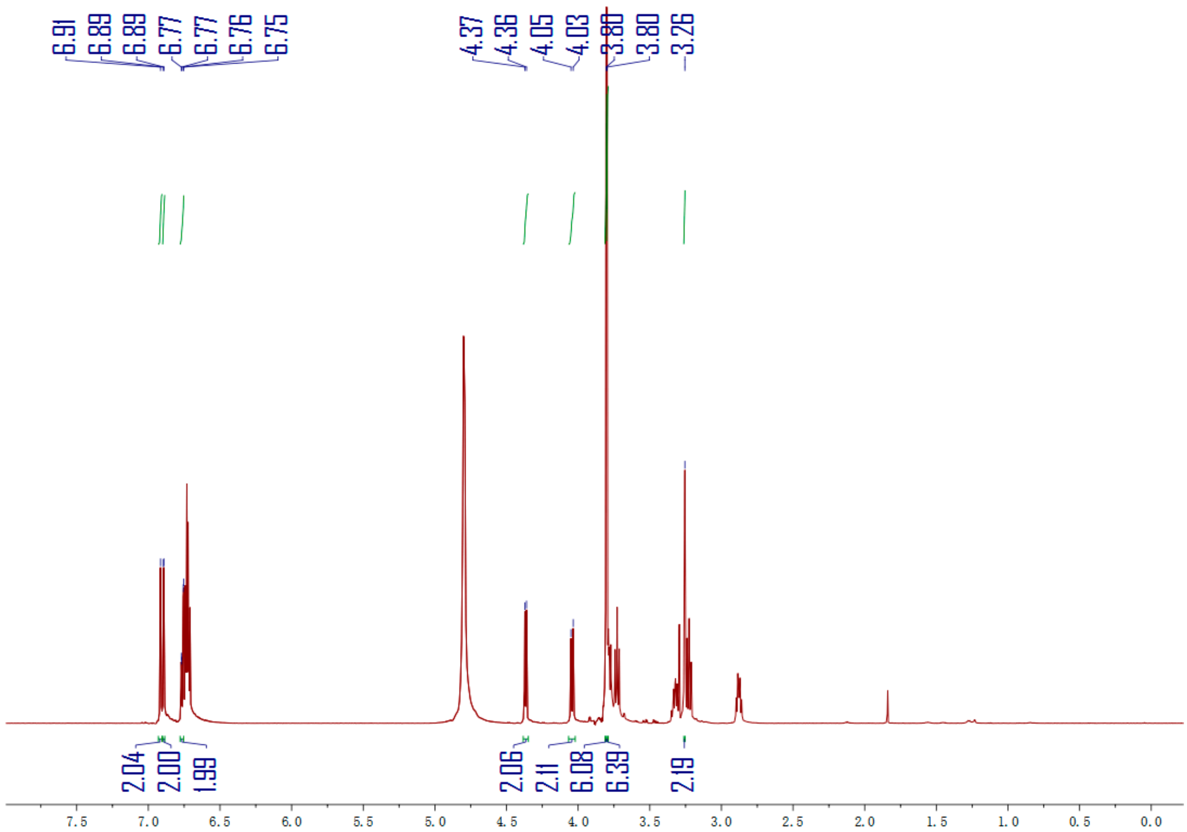

Figure S11. ^{1}H NMR of compound 5

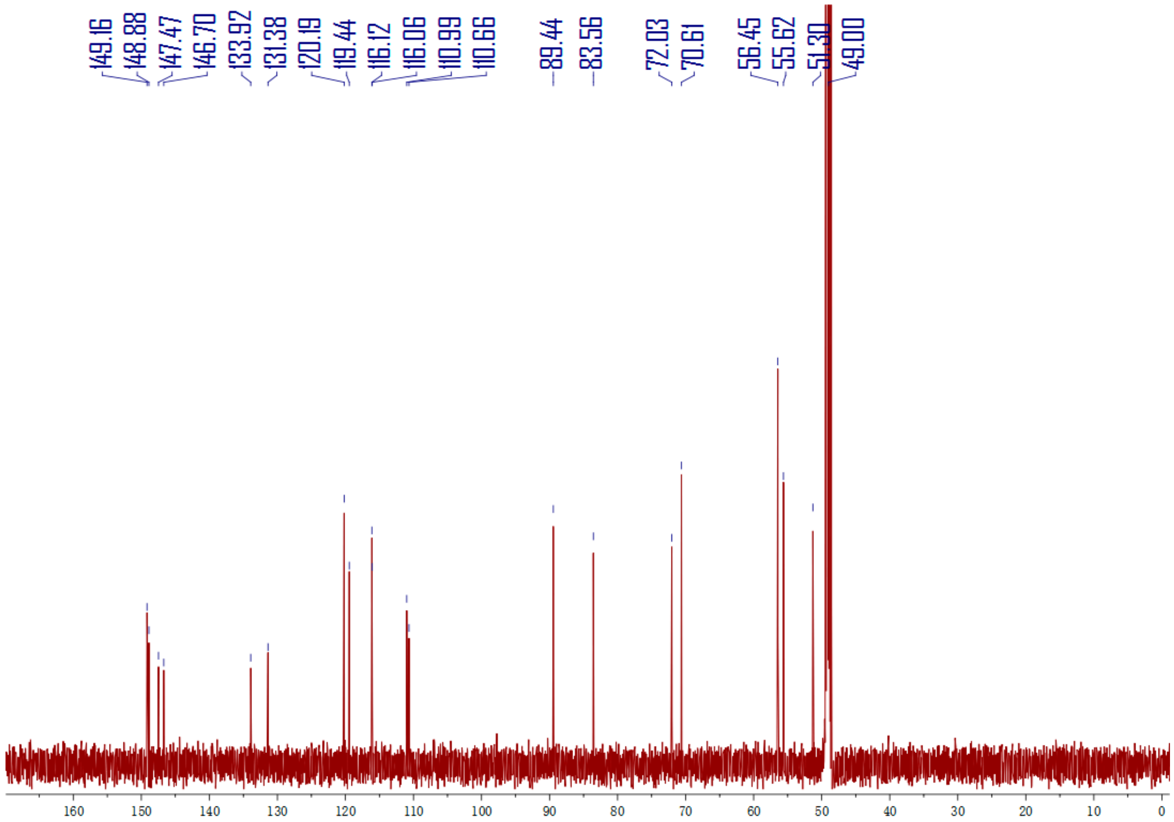

Figure S12. ^{13}C NMR of compound 5

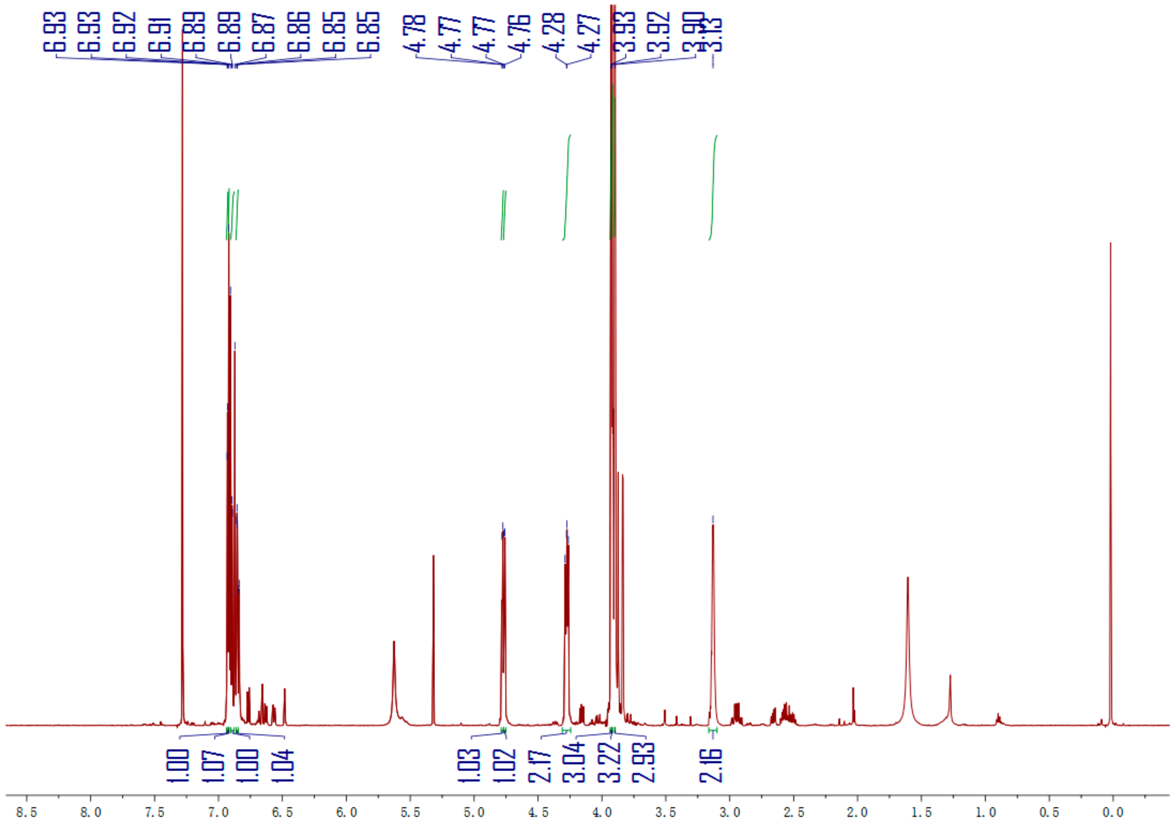

Figure S13. ^{1}H NMR of compound 6

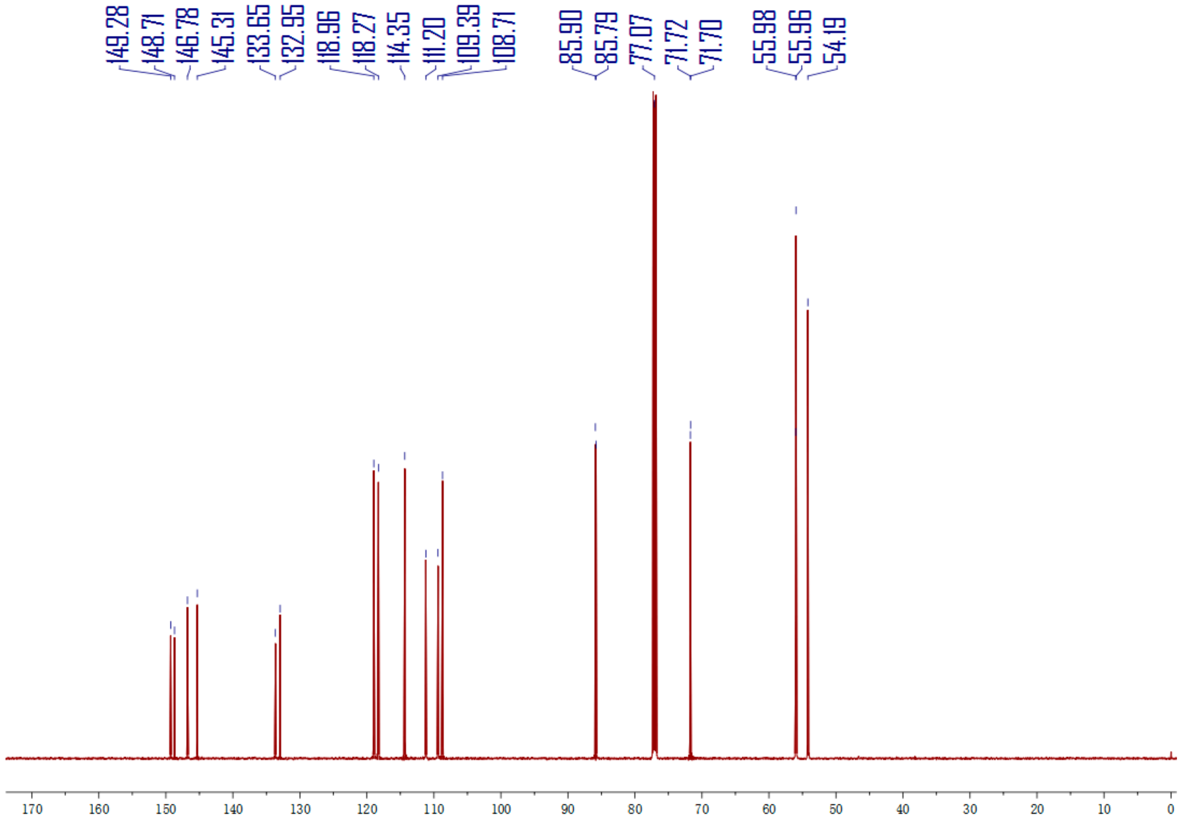

Figure S14. ^{13}C NMR of compound 6

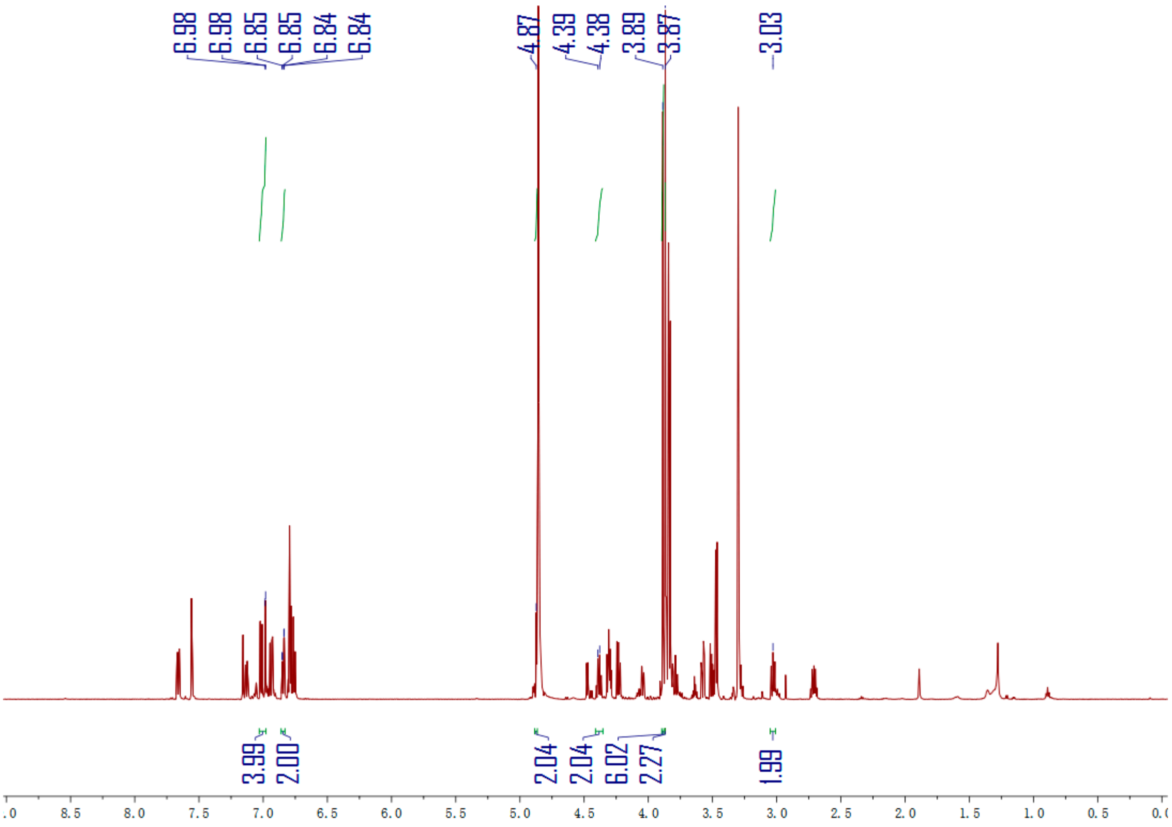

Figure S15. ^{1}H NMR of compound 7

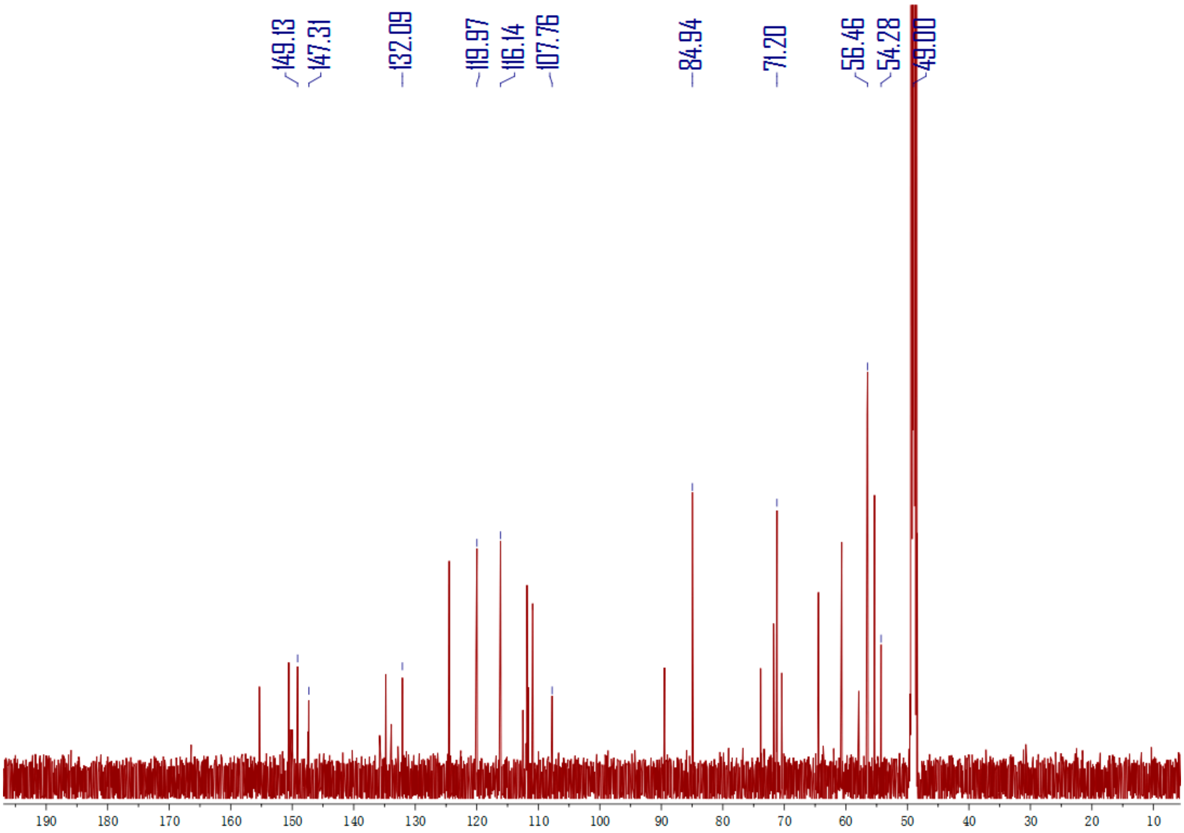

Figure S16. ^{13}C NMR of compound 7

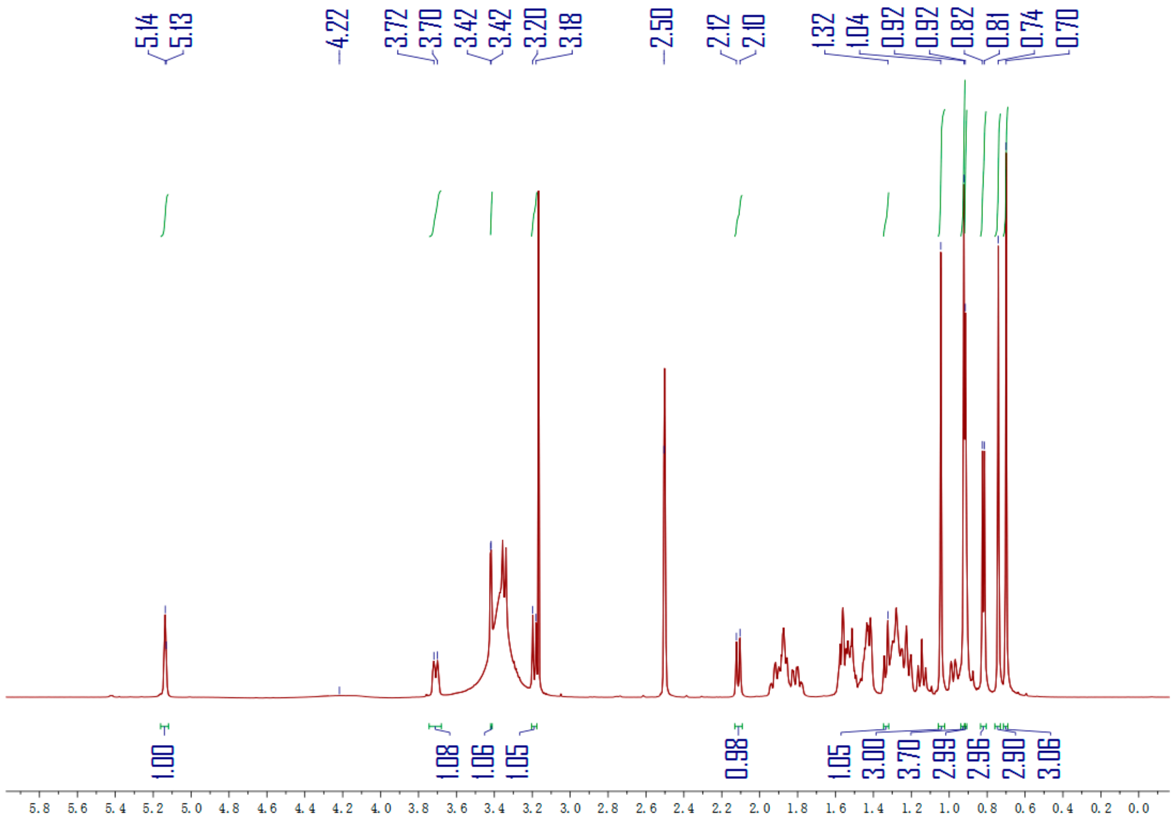

Figure S17. ^{1}H NMR of compound 8

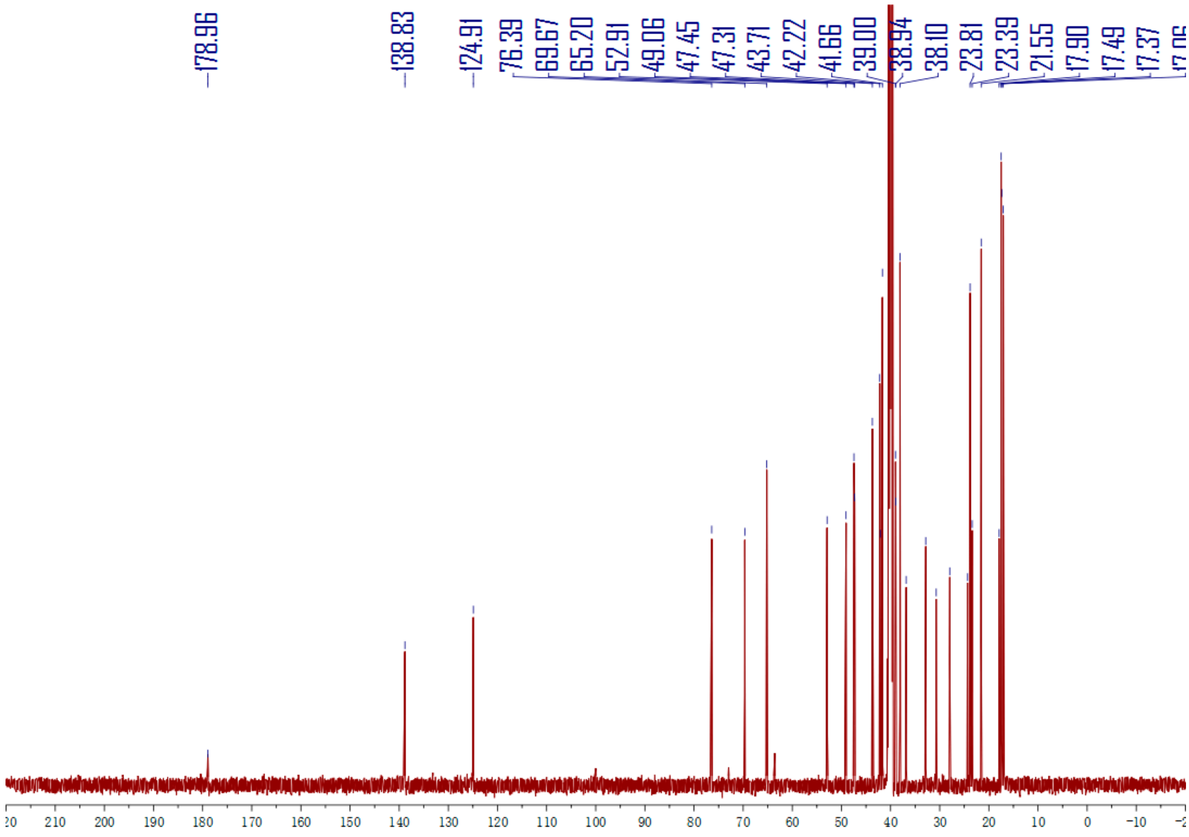

Figure S18. ^{13}C NMR of compound 8

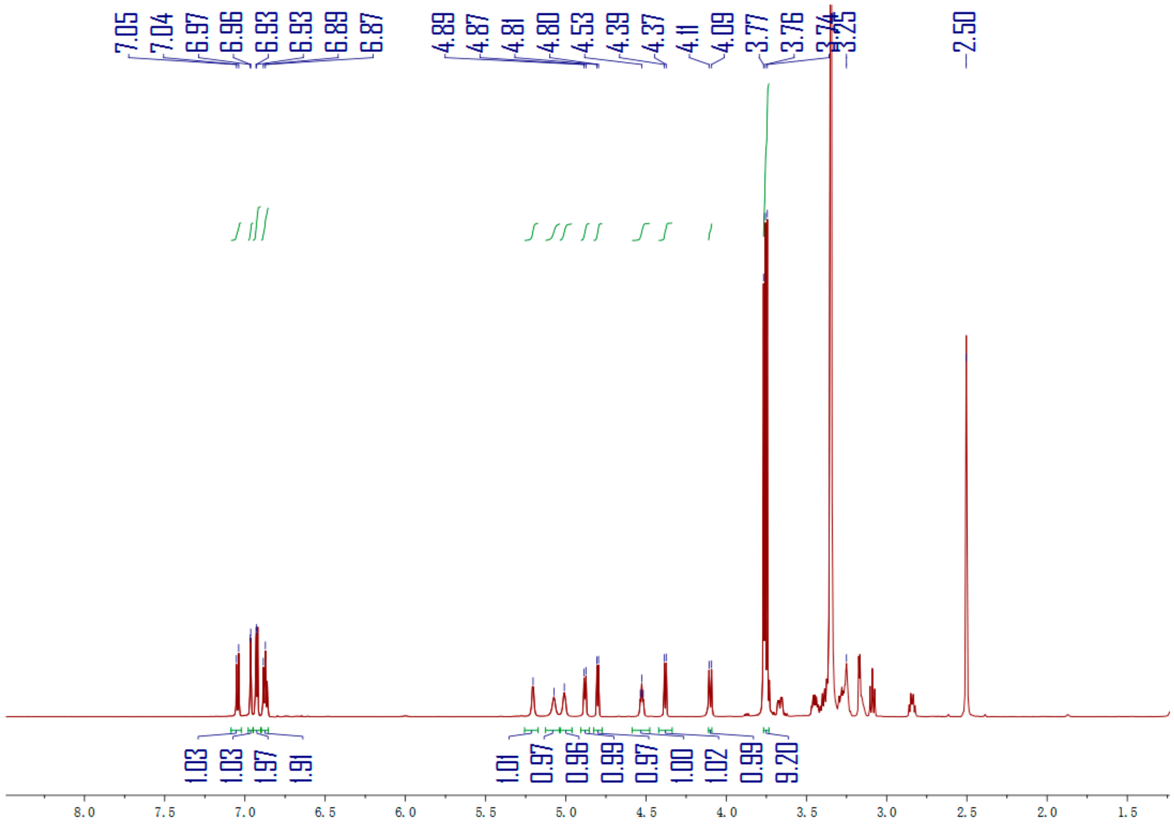

Figure S19. ^{1}H NMR of compound 9

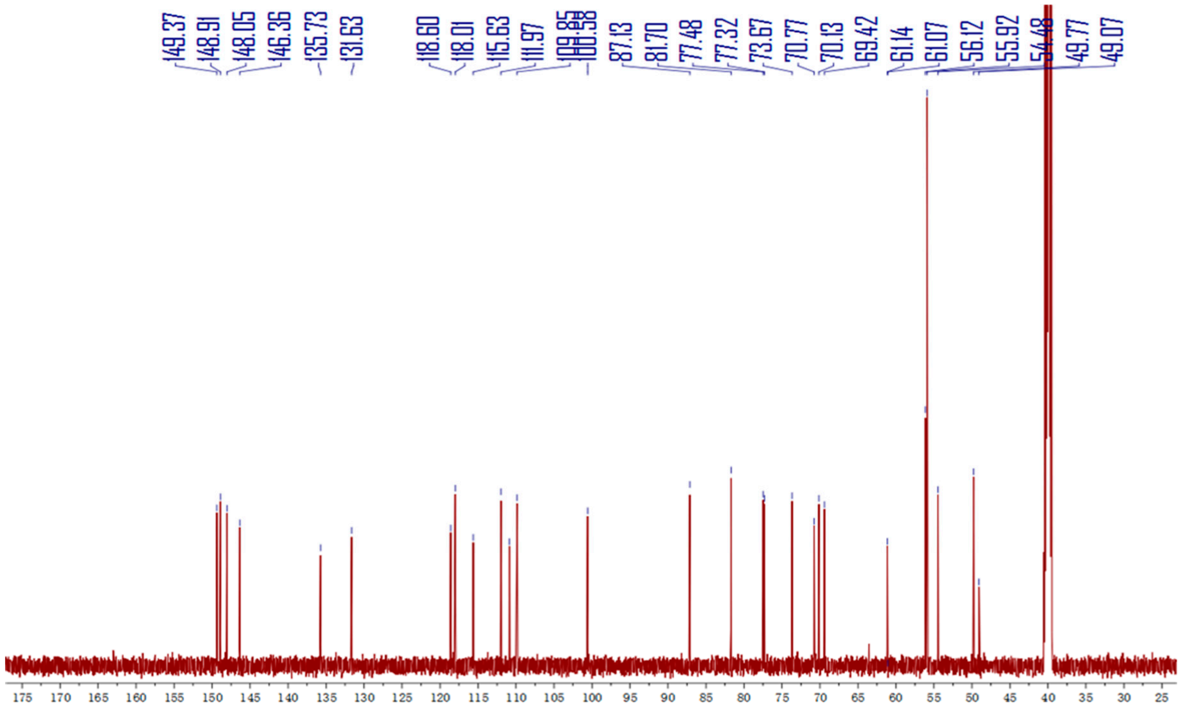

Figure S20. ^{13}C NMR of compound 9

7. Supplemental data of compounds 1–9

Table S10 ESI-MS data of compounds **1-9**

Compounds	Formula	Exact Mass	$[M-H]^-$	$[M+Na]^+$	$[M+H]^+$
compound **1**	$C_{21}H_{24}O_6$	372.41200	371.15045	395.14655	—
compound **2**	$C_{30}H_{48}O_3$	456.36034	455.35385	—	457.36767
compound **3**	$C_{30}H_{48}O_3$	456.36035	455.35355	479.34967	—
compound **4**	$C_{30}H_{48}O_3$	456.36029	455.35362	479.34964	—
compound **5**	$C_{20}H_{22}O_6$	358.38500	357.13495	381.13068	—
compound **6**	$C_{21}H_{24}O_6$	372.41166	371.15063	395.14661	—
compound **7**	$C_{20}H_{22}O_6$	358.39001	357.13476	381.13088	—
compound **8**	$C_{30}H_{48}O_5$	488.35017	487.34343	511.33936	—
compound **9**	$C_{27}H_{34}O_{11}$	534.21011	533.20365	557.19958	—

8. Supplemental ESI-MS Spectrum of compounds **1-9**

List of figures

Item	Subject
Figure. S21	$[M-H]^-$ of compound 1
Figure. S22	$[M+Na]^+$ of compound 1
Figure. S23	$[M-H]^-$ of compound 1
Figure. S24	$[M+Na]^+$ of compound 1
Figure. S25	$[M-H]^-$ of compound 1
Figure. S26	$[M+Na]^+$ of compound 1
Figure. S27	$[M-H]^-$ of compound 1
Figure. S28	$[M+Na]^+$ of compound 1
Figure. S20	$[M-H]^-$ of compound 1
Figure. S30	$[M+Na]^+$ of compound 1
Figure. S31	$[M-H]^-$ of compound 1
Figure. S32	$[M+Na]^+$ of compound 1
Figure. S33	$[M-H]^-$ of compound 1
Figure. S34	$[M+Na]^+$ of compound 1
Figure. S35	$[M-H]^-$ of compound 1
Figure. S36	$[M+Na]^+$ of compound 1
Figure. S37	$[M-H]^-$ of compound 1

Figure. S38	$[M+Na]^+$ of compound 1

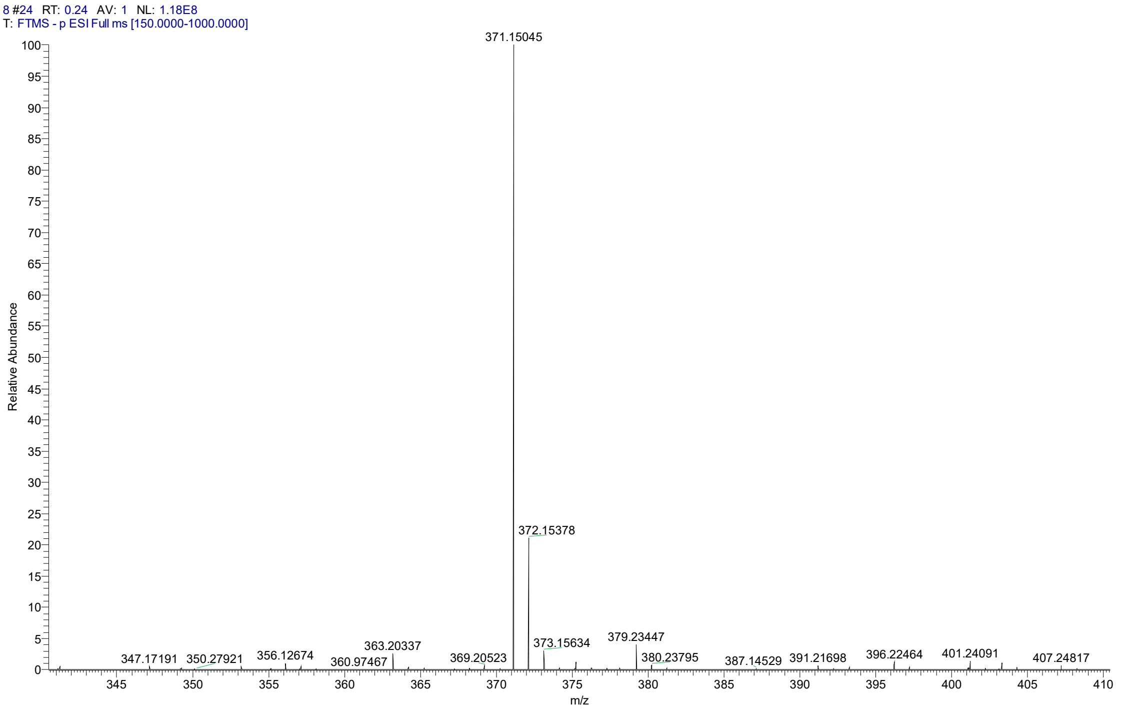

Figure S21. $[M{-}H]^-$ of compound 1

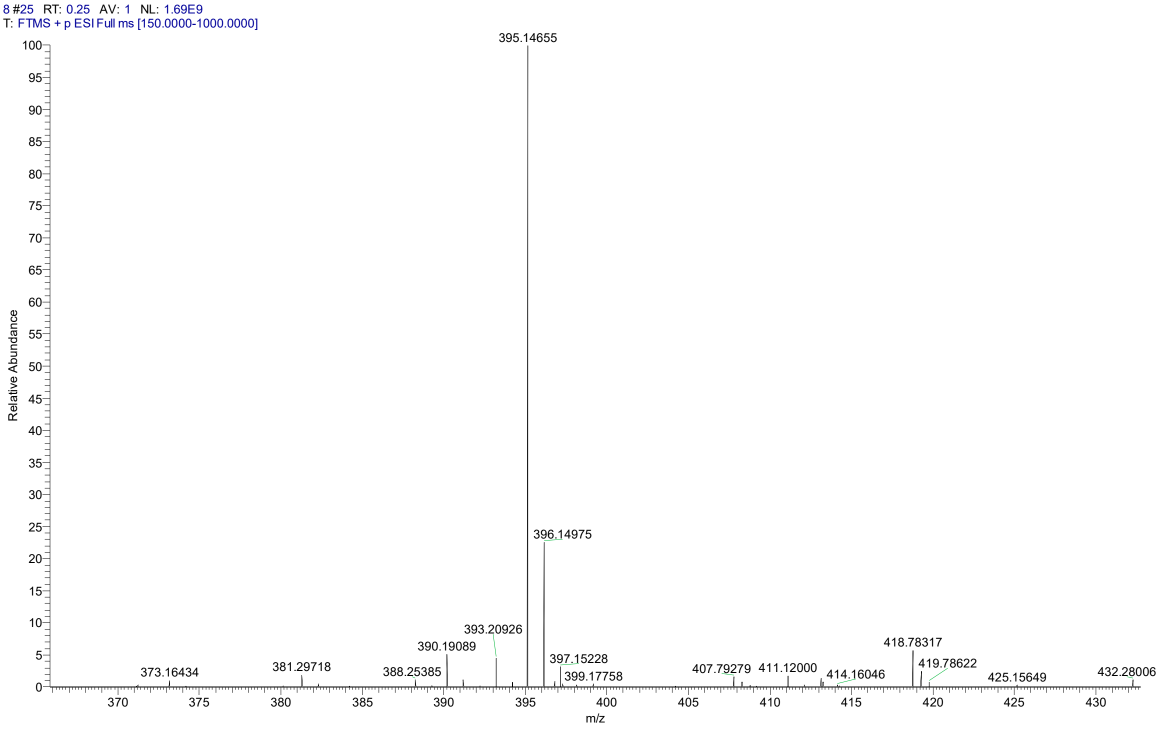

Figure S22. $[M+Na]^+$ of compound 1

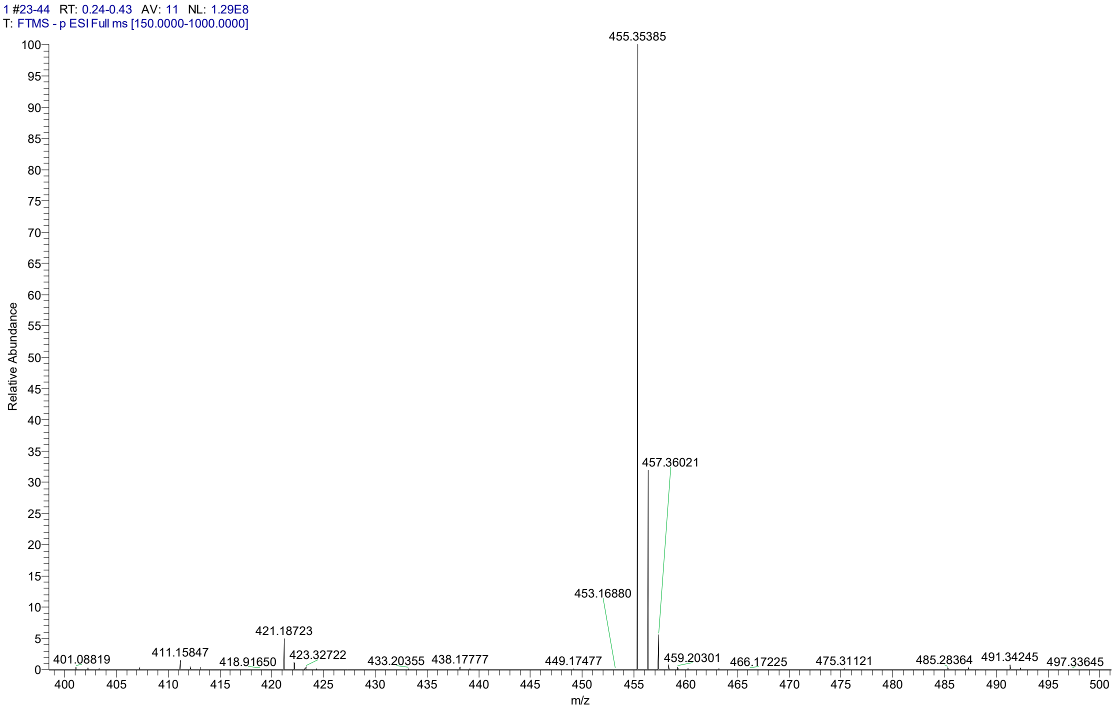

Figure S23. $[M-H]^-$ of compound 2

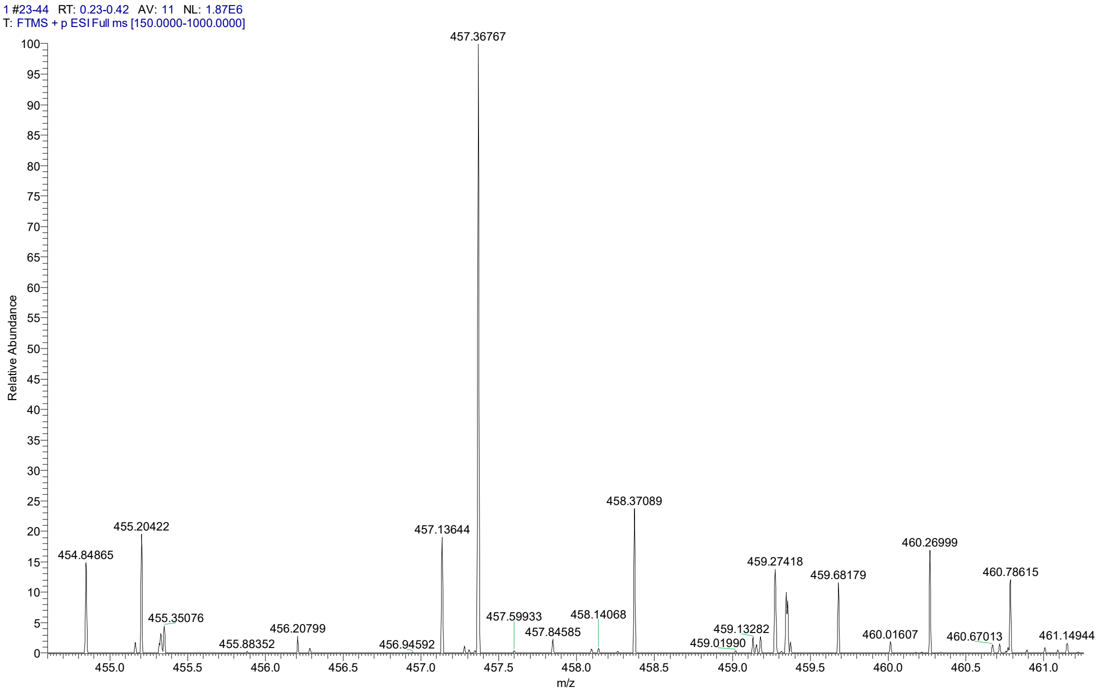

Figure S24. $[M+H]^+$ of compound 2

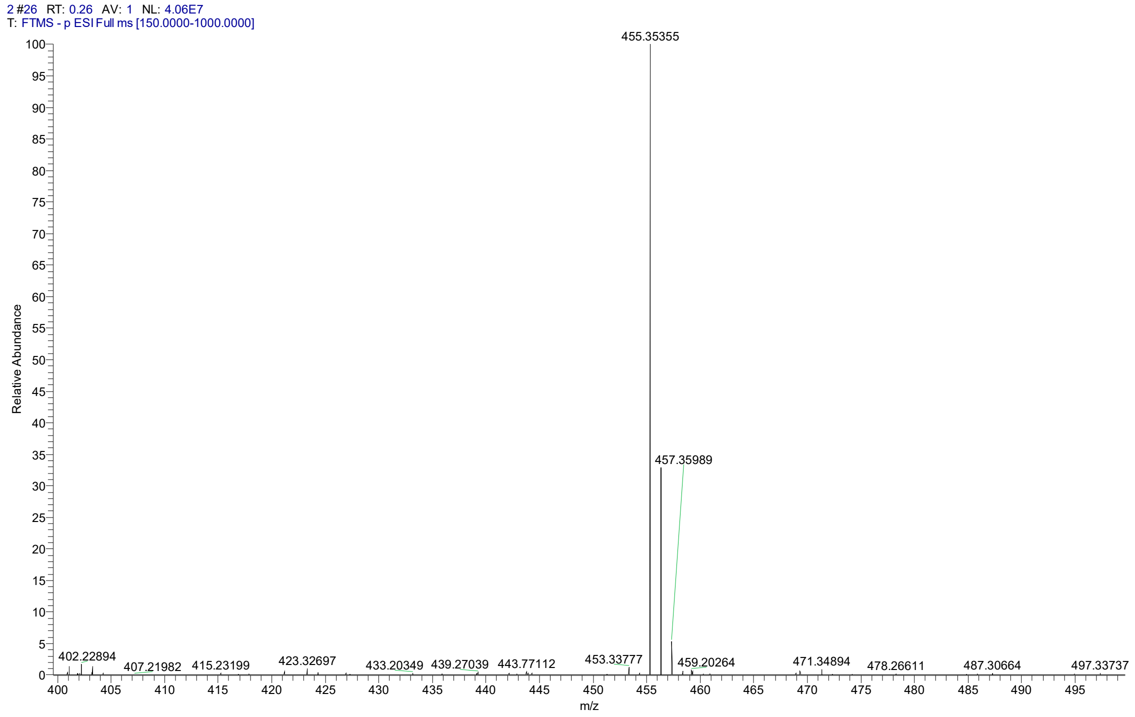

Figure S25. $[M-H]^-$ of compound 3

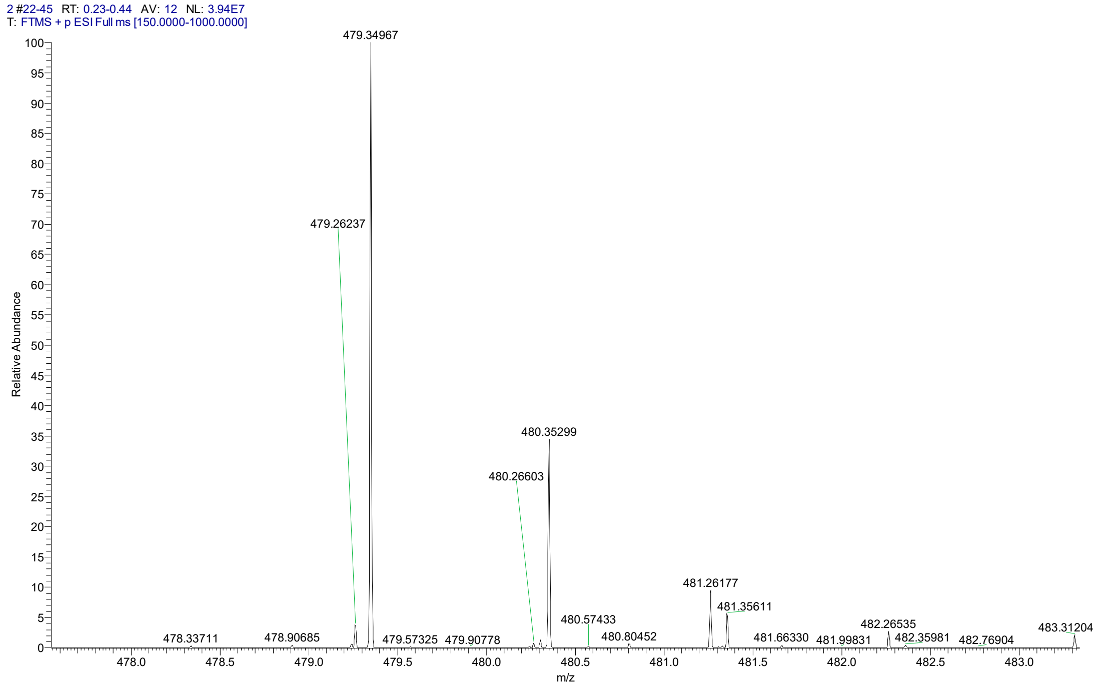

Figure S26. $[M+Na]^+$ of compound 3

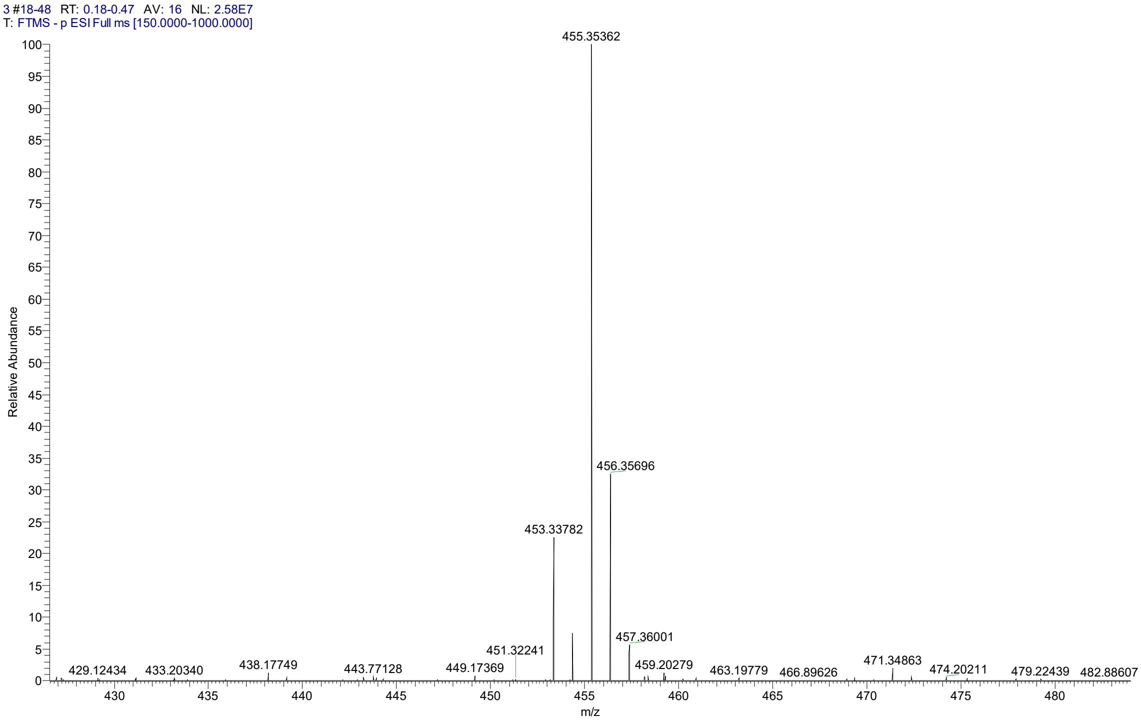

Figure S27. $[M{-}H]^-$ of compound 4

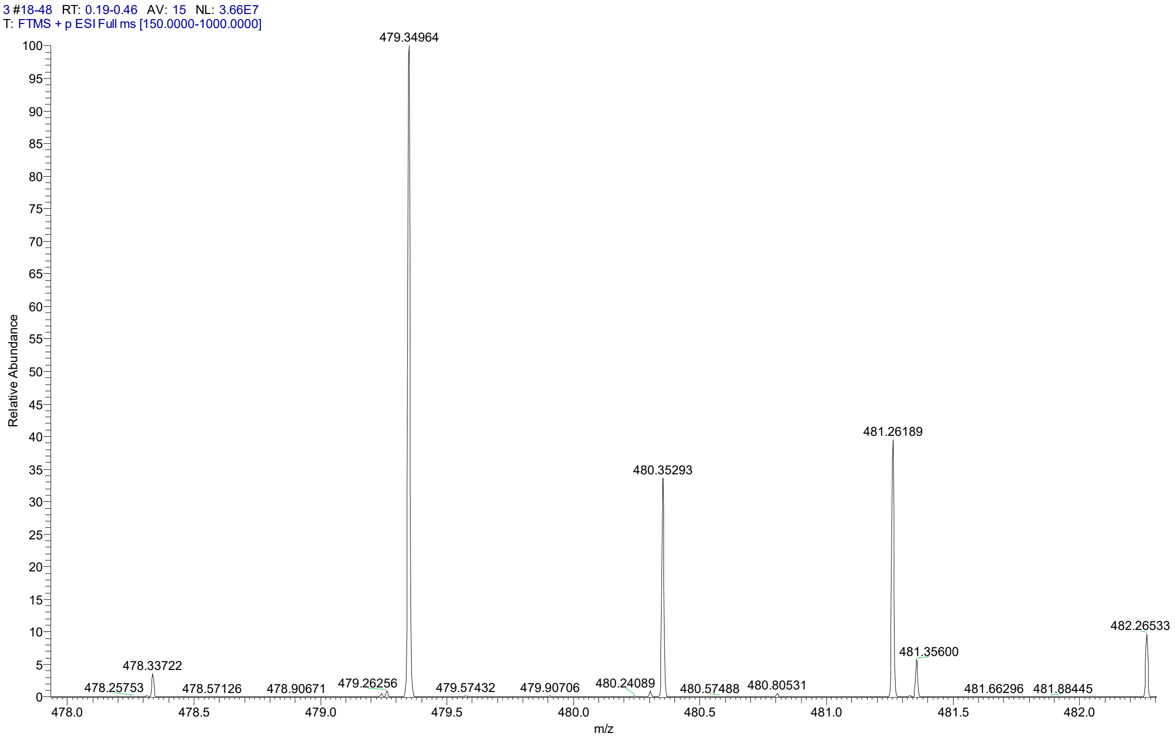

Figure S28. $[M{+}Na]^+$ of compound 4

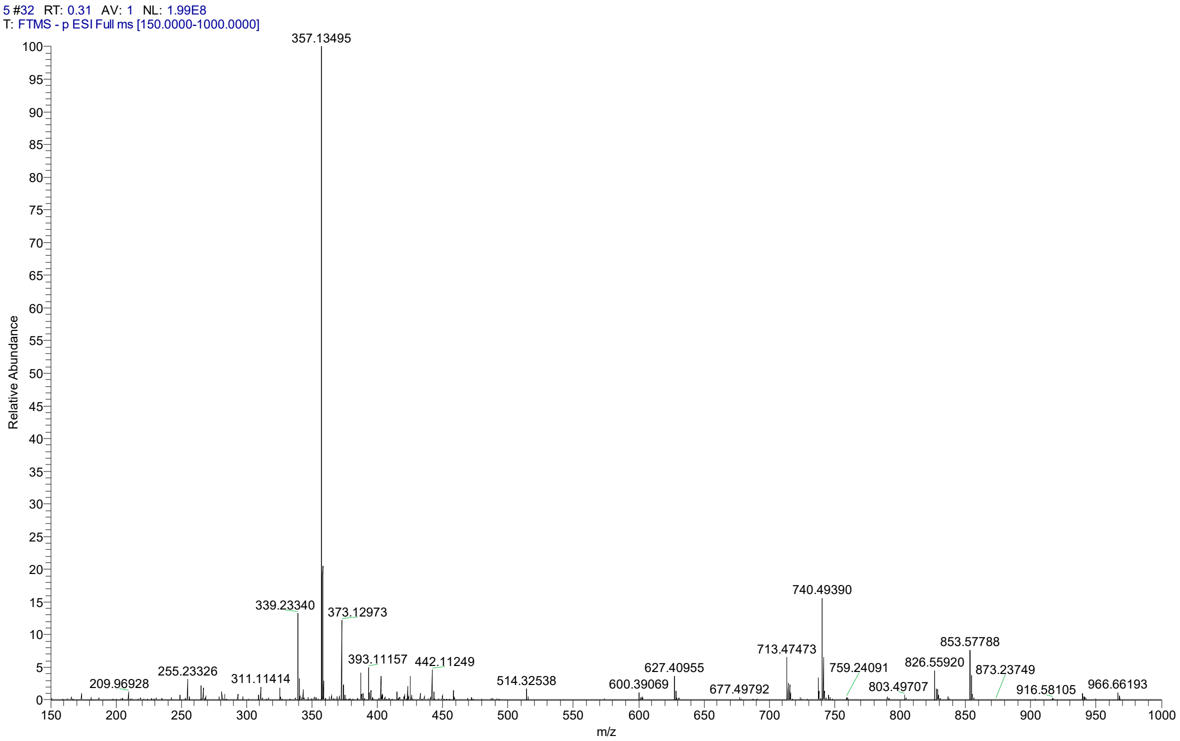

Figure S29. [M−H]$^{-}$ of compound 5

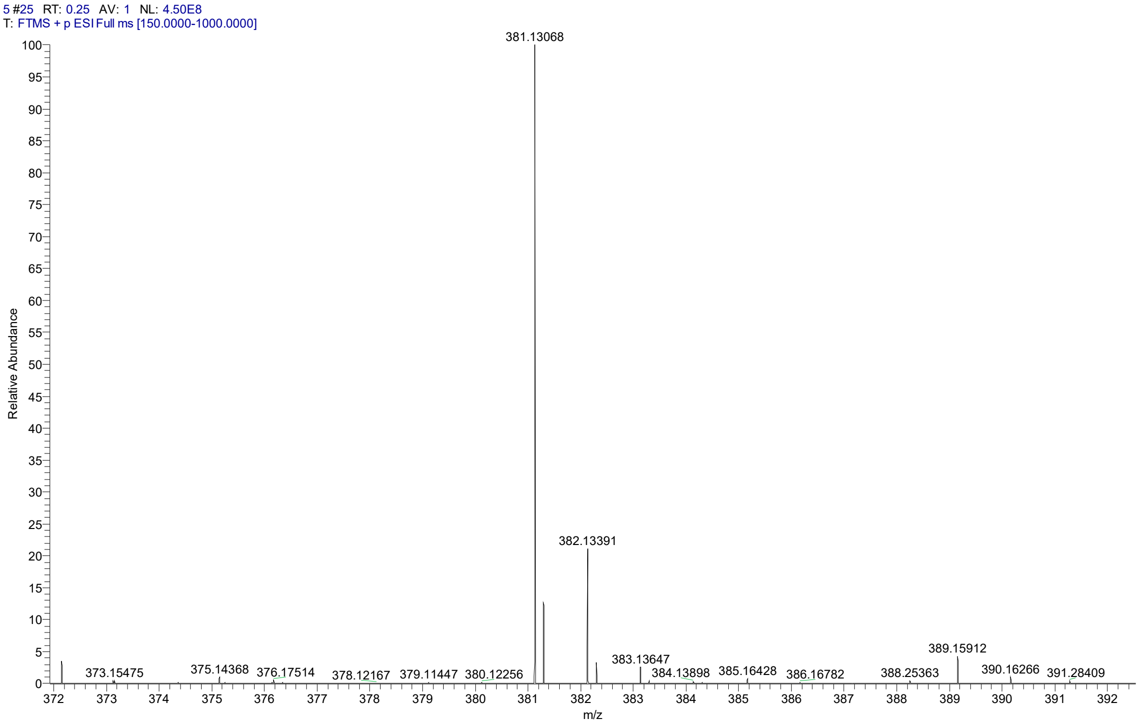

Figure S30. [M+Na]$^{+}$ of compound 5

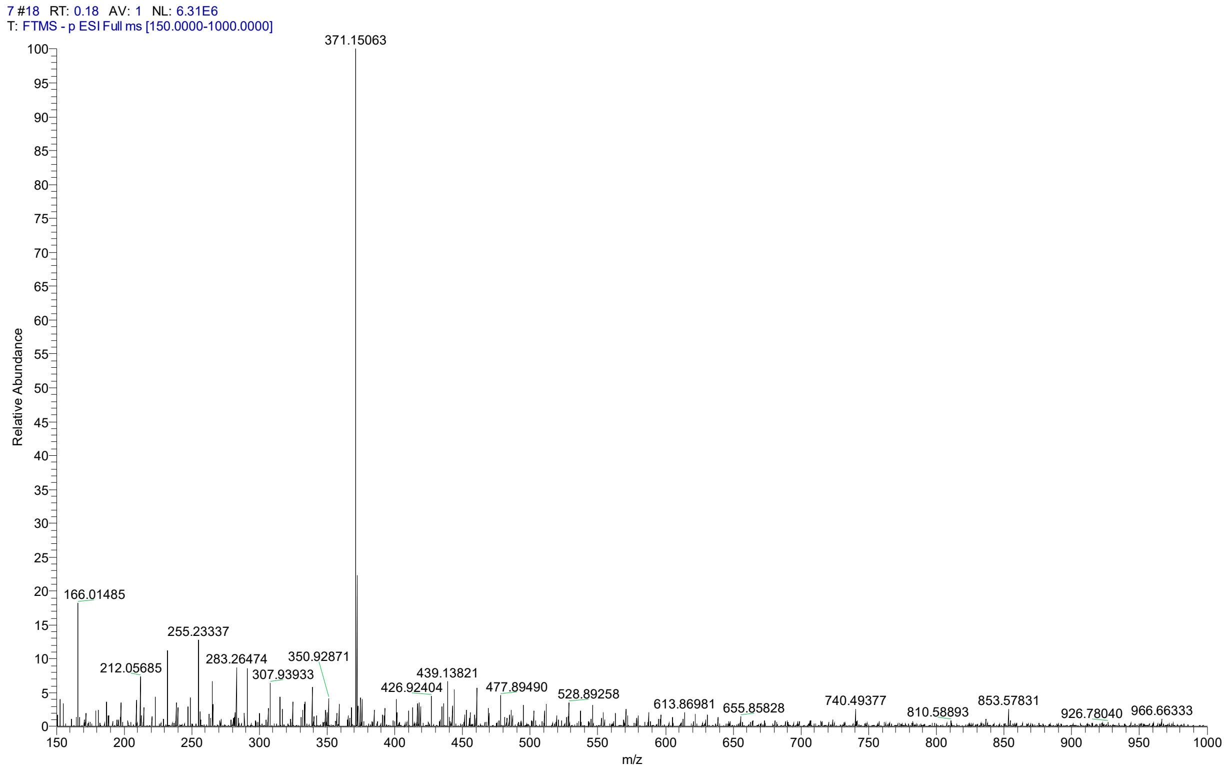

Figure S31. $[M-H]^-$ of compound 6

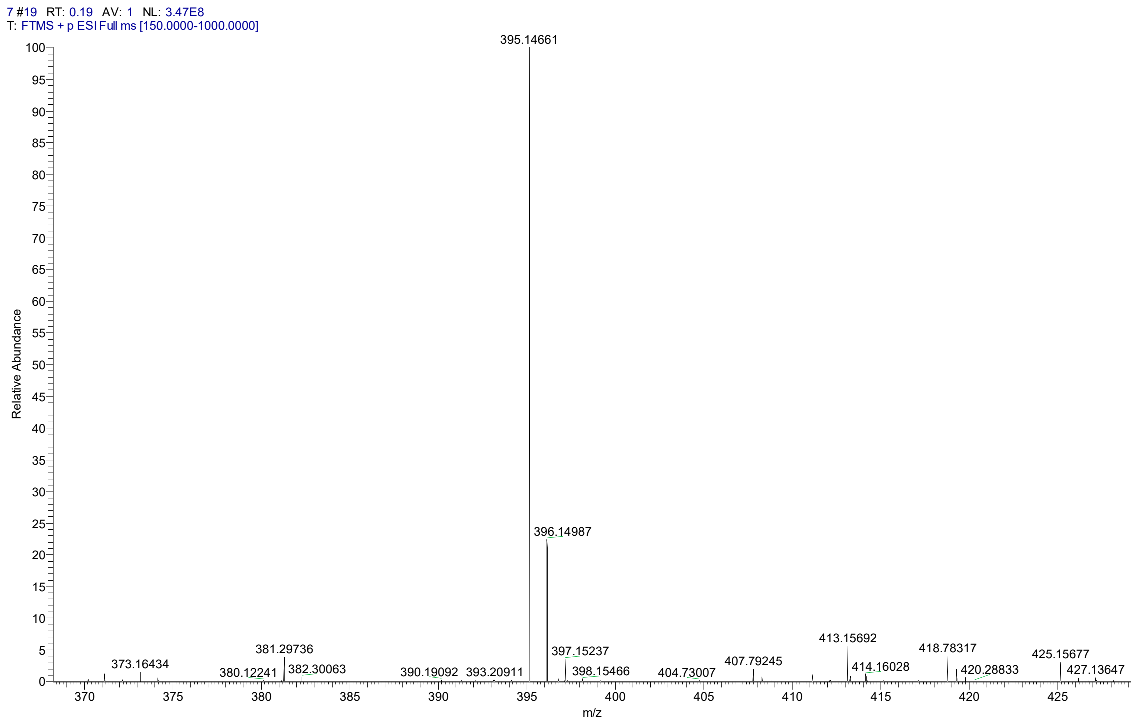

Figure S32. $[M+Na]^+$ of compound 6

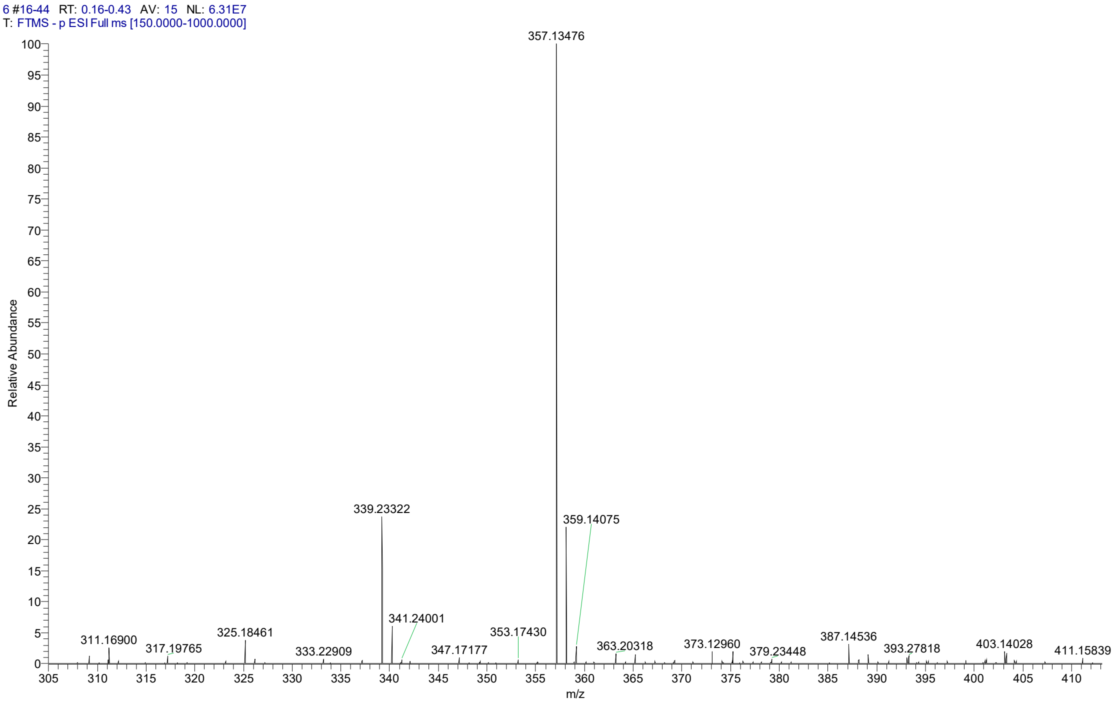

Figure S33. [M−H]$^{-}$ of compound 7

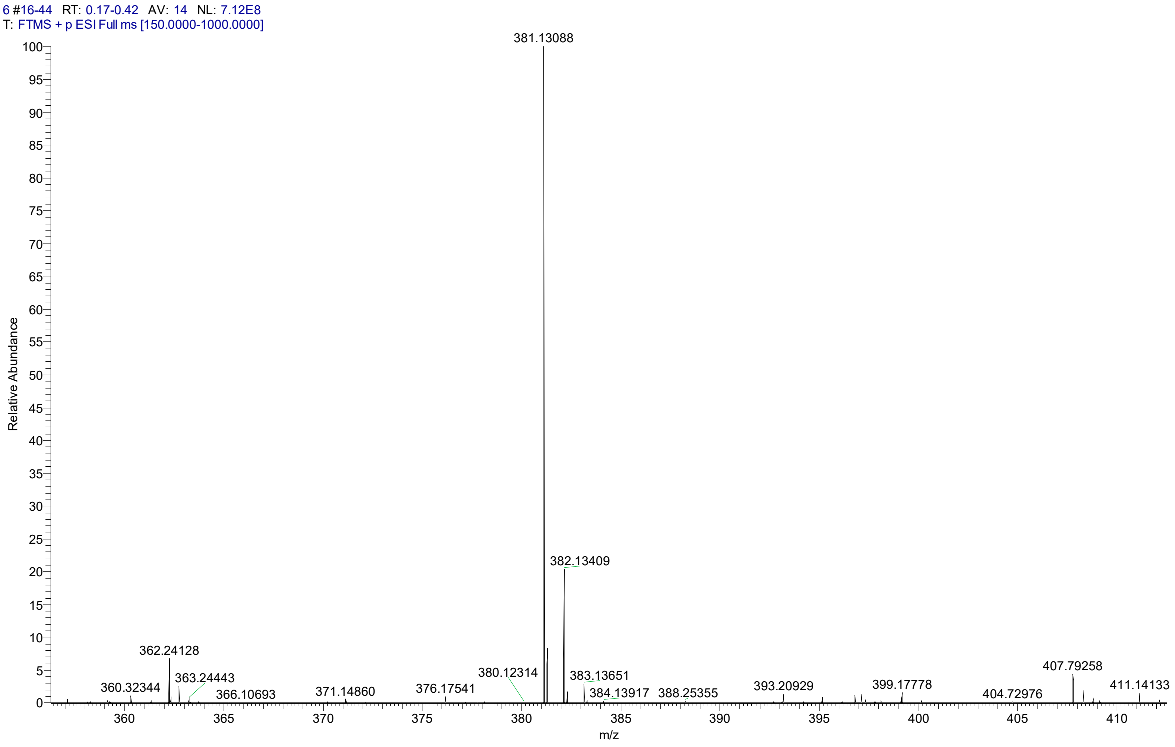

Figure S34. [M+Na]$^{+}$ of compound 7

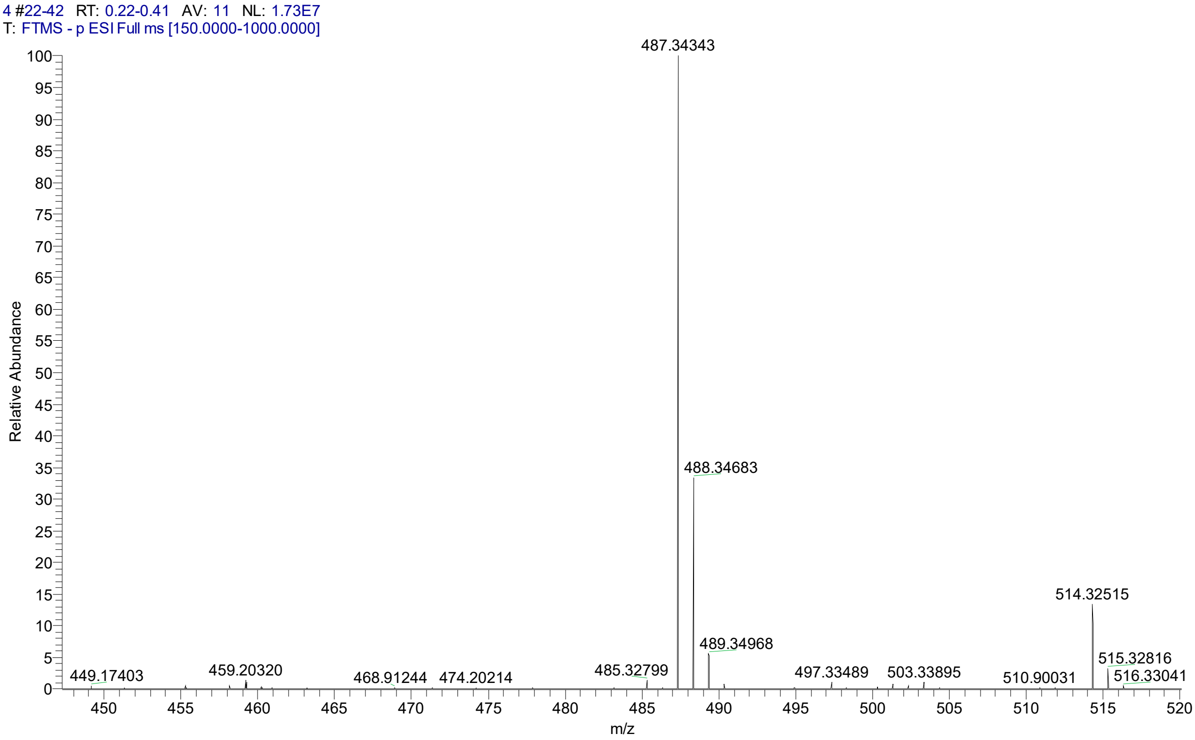

Figure S35. [M−H]⁻ of compound 8

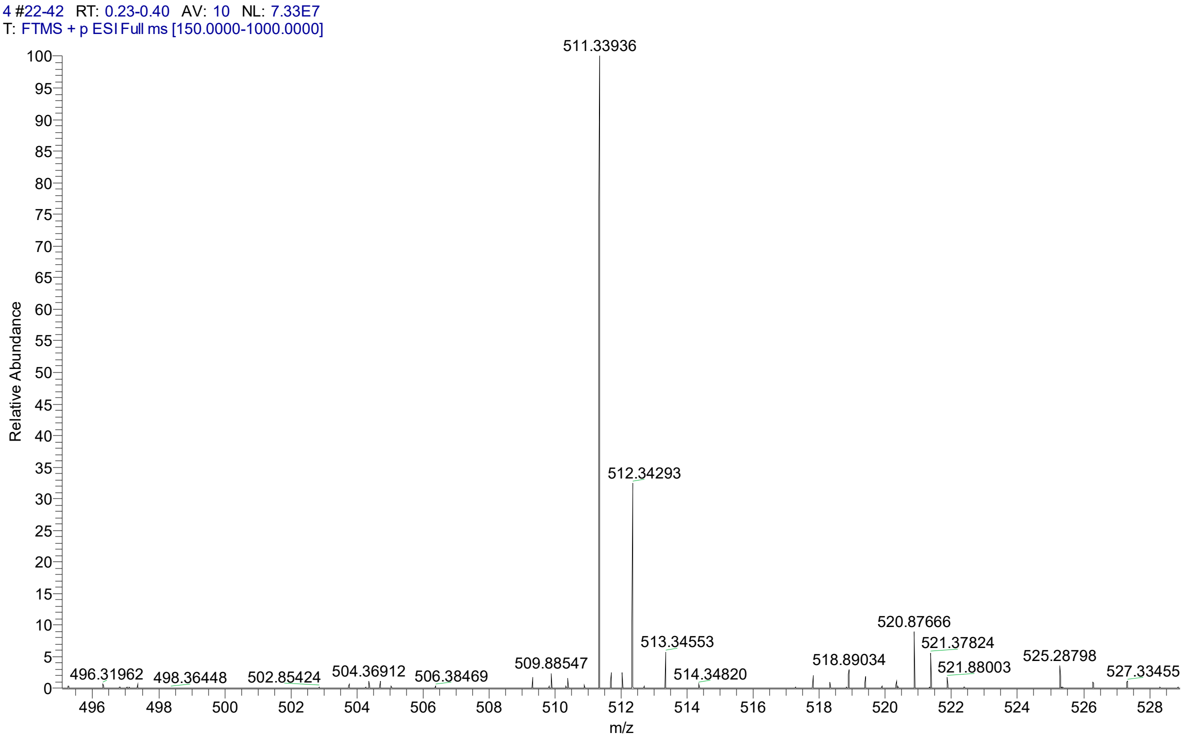

Figure S36. [M+Na]$^{+}$ of compound 8

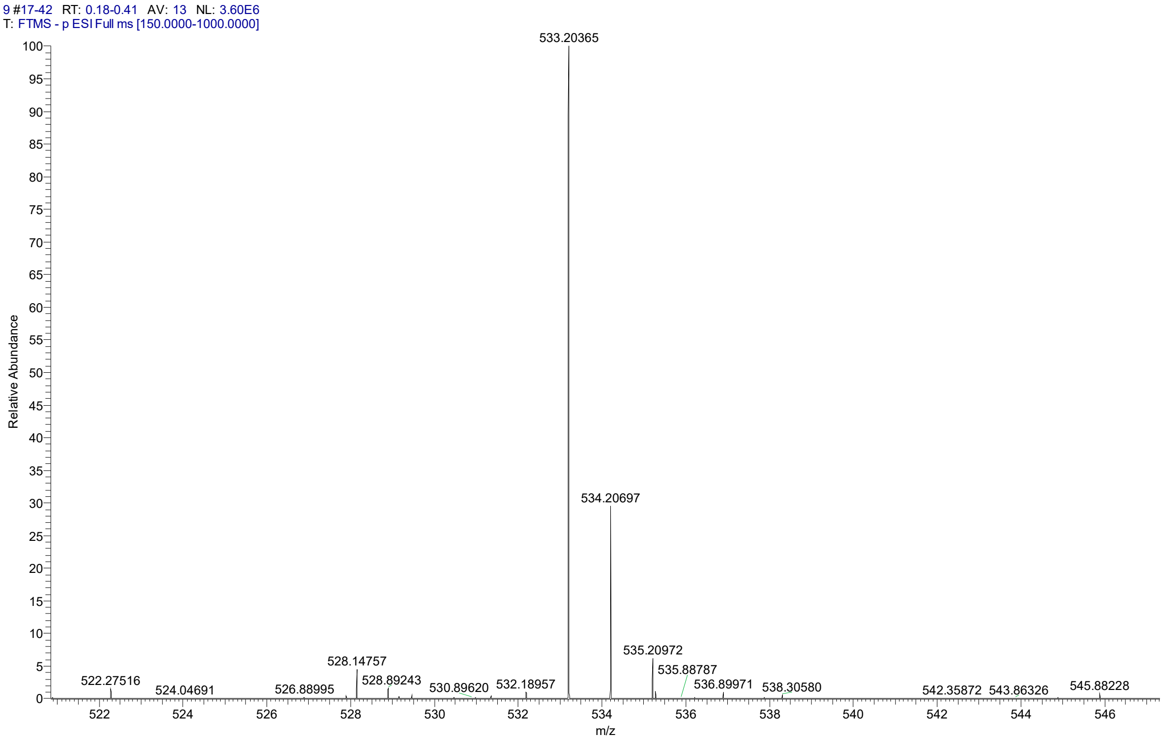

Figure S37. $[M-H]^-$ of compound 9

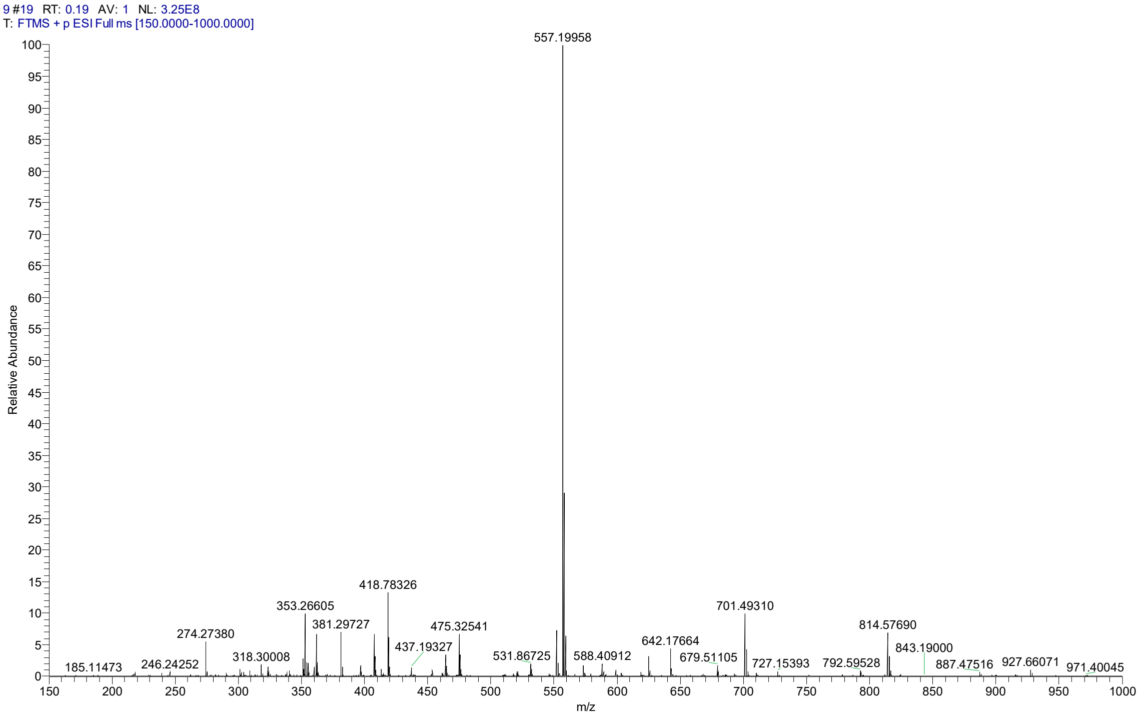

Figure S38. $[M+Na]^+$ of compound 9
